# Real-time NMR monitoring of HMF biotransformation by *Ectopseudomonas oleovorans* CECT 5344 R1D

**DOI:** 10.1007/s00253-026-13853-7

**Published:** 2026-05-07

**Authors:** Mar Gómez-Ortega, Sara Diego, Felipe Morales-Durán, Faustino Merchán, Rafael Blasco, Ana G. Neo, Carlos F. Marcos

**Affiliations:** 1 Department of Biochemistry, School of Veterinary Sciences, Universidad de Extremadura, Cáceres, 10003 Spain; 2 Laboratory of Bioorganic Chemistry & Membrane Biophysics (L.O.B.O.), Departamento de Química Orgánica E Inorgánica, Universidad de Extremadura, Cáceres, 10003 Spain

**Keywords:** Biorefinery, Biodetoxification, Circular economy, HMF, HMFCA, Nuclear magnetic resonance, Biomass

## Abstract

**Abstract:**

This research identifies *Ectopseudomonas oleovorans* CECT 5344 R1D as a novel and highly efficient biocatalyst for the sustainable production of 5-hydroxymethyl-2-furancarboxylic acid (HMFCA). By employing benchtop Nuclear Magnetic Resonance (NMR) spectroscopy with a recirculating flow system, we achieved real-time monitoring and a detailed stoichiometric characterisation of the 5-hydroxymethylfurfural (HMF) detoxification pathway. This work contributes to the specialised field of real-time metabolic tracking of living biocatalysts, a niche where in situ NMR data is still scarce compared to end-point analytical methods. Our findings demonstrate that this strain, while not utilising HMF for biomass growth, maintains exceptional metabolic stability and quantitative molar yields over 125 h of operation, requiring significantly lower cell densities than other reported *Pseudomonas* strains. The in-line NMR setup enabled the definitive identification of 2,5-bis(hydroxymethyl)furan (BHMF) as a transient intermediate, clarifying previous metabolic misidentifications and revealing a highly specific, non-consuming “cell factory” behaviour. Furthermore, the system allowed for the assessment of bacterial tolerance thresholds and the exploration of the enzymatic substrate scope against other environmentally relevant aldehydes. This work underscores the synergy between a robust, living biocatalyst and a precise monitoring platform, providing an optimised workflow for bioprocess development in sustainable aldehyde valorisation.

**Key points:**

• *Real-time monitoring of a microbial process via in-line benchtop NMR.*

• *Versatile biodetoxification of various natural and synthetic aldehydes.*

•* Green chemistry alternative to petrochemicals using renewable feedstocks.*

**Graphical abstract:**

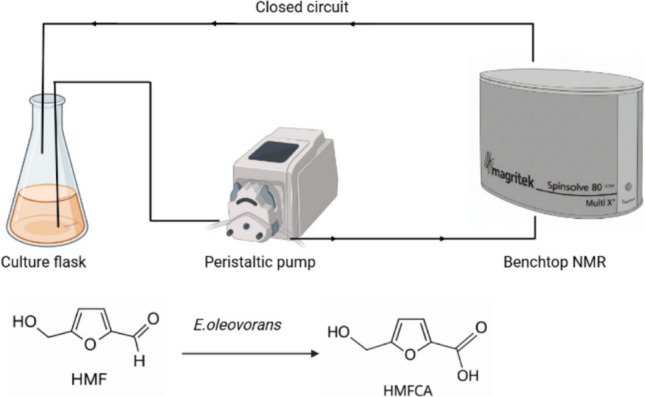

**Supplementary information:**

The online version contains supplementary material available at 10.1007/s00253-026-13853-7.

## Introduction

The increasing global demand for chemicals and materials, largely satisfied by the petrochemical industry, has intensified concerns about environmental impact and the long-term sustainability of relying on finite fossil fuel resources. This dependence highlights an urgent need to transition towards sustainable chemical production routes that utilise renewable resources. Lignocellulosic biomass, an abundant and renewable carbon source derived from wood and agricultural residues, shows great promise as a feedstock for generating a diverse range of valuable chemicals and materials. This offers a compelling pathway to mitigate the environmental challenges associated with conventional petrochemical processes and actively contribute to a circular economy.

Among the wide array of biomass-derived compounds, 5-hydroxymethylfurfural (HMF) stands out as a key platform chemical, having garnered significant research attention (Li and Zong 2022; van Putten et al. 2013). This versatile molecule, formed through the dehydration of biomass-derived carbohydrates, features a unique chemical structure comprising a furan ring, a hydroxyl group, and a formyl group (Yu and Tsang 2017). This trifunctional nature enables HMF to undergo a broad spectrum of chemical transformations, positioning it as a vital intermediate in the synthesis of numerous high-value products. The strategic significance of HMF is further underscored by its designation as a top platform chemical by the U.S. Department of Energy, emphasising its central contribution to the emerging biorefinery paradigm and the development of a bio-based economy (Bozell and Petersen 2010; Chen et al. 2024). The consistent focus on HMF as a fundamental building block across multiple research domains demonstrates a strong consensus within the scientific community regarding its capacity to facilitate the shift towards more sustainable chemical production methodologies.

Biotransformation, utilising the catalytic capabilities of biological systems, offers a green and sustainable approach for converting HMF into higher-value products. This approach offers several advantages, including high selectivity and the ability to perform reactions under mild conditions, thereby reducing energy consumption and the generation of hazardous byproducts. A particularly relevant biotransformation in the context of HMF-based biorefinery platforms is the conversion of HMF into 5-hydroxymethyl-2-furancarboxylic acid (HMFCA). This valuable intermediate is produced by the selective oxidation of HMF’s aldehyde group, while its alcohol group remains intact. HMFCA serves as a crucial building block for the synthesis of various biodegradable polyesters (Hao et al. 2020; Todea et al. 2019), is a precursor of terephthalic acid (PTA) (Pacheco and Davis 2014), and has been used as a starting material in the synthesis of potent IL-2 inhibitors (Braisted et al. 2003). Furthermore, HMFCA is a key intermediate in the full oxidation pathway toward 2,5-furandicarboxylic acid (FDCA). FDCA is currently the most commercially significant product derived from HMF, as it is the primary monomer for the production of poly(ethylene 2,5-furandicarboxylate) (PEF), a bio-based alternative to petroleum-derived polyethylene terephthalate (PET) and currently the only commercialised polymer involving this metabolic pathway (Eerhart et al. 2012; Motagamwala et al. 2018).

While the bacterial biotransformation of HMF has gained increasing attention, reports on microorganisms capable of performing this conversion without assimilating the resulting metabolites remain relatively scarce. Most research to date has focused on HMF-assimilating bacteria, which utilise this compound as a carbon source for growth. In contrast, there are fewer reports on specialised biocatalysts that exclusively biotransform HMF into industrially relevant secondary metabolites such as HMFCA, with high molar yields and no further consumption.

Among the microorganisms explored for this purpose, the genus *Pseudomonas* stands out for its metabolic versatility. These bacteria possess a broad enzymatic repertoire that enables them to efficiently transform a variety of organic compounds, including furan derivatives. Specifically, strains such as *P. putida* KT2440 (Xu et al. 2020), *P. aeruginosa* PC-1 (Pan et al. 2020), *P. taiwanensis* VLB120 (Lechtenberg et al. 2024), *P. rhodesiae* NL2019 (Liu et al. 2023), *P. putida* S12 (Hsu et al. 2020) and *Pseudomonas sp.* MB04B (Liu et al. 2025) have exhibited remarkable capabilities in converting HMF into HMFCA and other valuable products such as 2,5-furandicarboxylic acid (FDCA) (Table 1). The natural occurrence of *Pseudomonas* in environments with decaying plant materials, where furan compounds are present, suggests an evolutionary adaptation that provides this genus with the necessary metabolic pathways for metabolising such substrates (Nichols et al. 2012; Parales and Harwood 2002). Consequently, *Pseudomonas* represents a promising platform for developing robust biocatalytic processes for HMF valorisation.

**Table 1:** Comparison of HMF performance between *E. oleovorans* CECT 5344 R1D and other microbial platforms

Strain	Biomass (OD_600_)^a^	Supp. medium^b^	HM (mM)	Major product	Yield (%)	Q_P_^c^	Reference
*E. oleovorans* CECT 5344 R1D	0.13	No	10	HMFCA	> 98	0.71	This work
*P. putida* KT2440	25	No	160	HMFCA	96.8	12.9	(Xu et al. 2020)
*P. aeruginosa* PC-1	10^d^	Yes	100	HMFCA	90	15	(Pan et al. 2020)
*P. taiwanensis* VLB120	0.5	Yes	10	FDCA	> 90	1.13	(Lechtenberg et al. 2024)
*P. rhodesiae* NL2019	n.d.^e^	Yes	280	HMFCA	94.8	33.2	(Liu et al. 2023)
*P. putida* S12	100	Yes	100	FDCA	96	2	(Hsu et al. 2020)
*Pseudomonas sp.* MB04B	100	No	100	FDCA	97	8	(Liu et al. 2025)

^a^OD_600_ values provided in each study. *n.d.* not determined. ^b^Supplemented medium refers to whether the biotransformation was performed in a mineral medium alone (“No”) or with additional assimilable carbon sources (“Yes”). ^c^*Q*_*P*_, volumetric productivity calculated as mmol/L·h (Δ[HMF]/Δt). ^d^Corresponds to 5 mg/mL CDW. ^e^10 mg/mL CDW

Building on this versatility, our attention has focused on *Ectopseudomonas oleovorans* CECT 5344 (formerly *Pseudomonas pseudoalcaligenes* CECT 5344). This bacterium, isolated from sludge in the Guadalquivir River, has been the focus of studies for its ability to use cyanide as its sole nitrogen source (Luque-Almagro et al. 2005). Genome sequencing of *E. oleovorans* CECT 5344 initially indicated the strain’s potential to degrade furanic compounds. This potential was subsequently confirmed by our previous investigations into its biotransformation of furfural and HMF (Luque‐Almagro et al. 2013). Specifically, we found that the R1D strain of *E. oleovorans* CECT 5344 was unable to assimilate HMF but was capable of transforming it into an unknown aromatic compound and a minor amount of a substance tentatively identified as 2,5-furandicarboxylic acid (FDCA) (Igeño et al. 2019). Confirming the precise identity of these compounds and conducting a detailed study of this biotransformation are crucial steps for its successful implementation within a biorefinery platform aimed at producing value-added furan derivatives.

The observed biotransformation of HMF is possibly facilitated by the presence of non-specific alcohol and aldehyde dehydrogenases (Chang et al. 2023; Koopman et al. 2010). HMF oxidation may provide the reducing power necessary to sustain bacterial viability under carbon-limited conditions while simultaneously mitigating its toxic effects. This toxicity typically stems from the high reactivity of the aldehyde moiety toward cellular nucleophiles, such as protein thiols and amino groups (Lopachin and Gavin 2014).

**Fig. 1:**
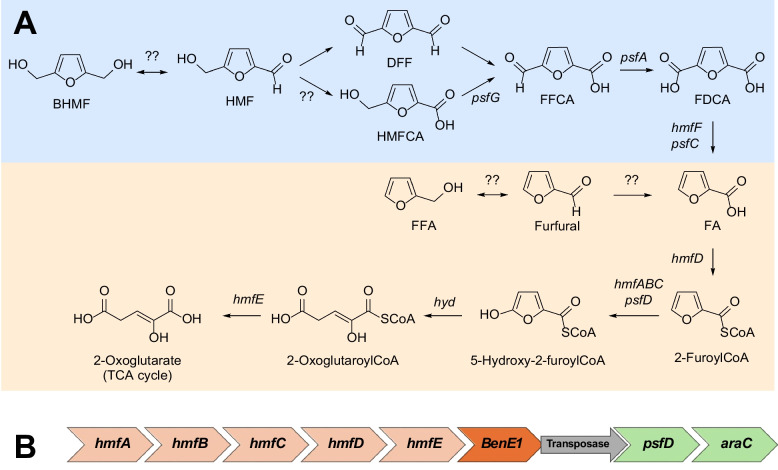
Metabolic and genetic context of HMF biotransformation. **A** Proposed metabolic pathways for the degradation of HMF (blue background) and furfural (orange background) in *Ectopseudomonas oleovorans* CECT 5344 R1D. Both converge at 2-furoic acid via the *hmfABCDE* gene cluster. Double question marks indicate steps where specific enzymes have yet to be identified. **B** Genetic organisation of the *hmf* operon in the parental strain CECT 5344

A thorough understanding of the specific reaction pathways for the oxidation of furfural, HMF and other furans is crucial for optimising the biotransformation process. The catabolism of furfural and HMF proceeds through an oxidative pathway, ultimately converging into furoic acid (FA), which is subsequently converted into the central metabolite 2-oxoglutarate (Fig. 1). By analogy to the well-known assimilatory pathway of toluene and xylenes encoded by the TOL plasmid in *Pseudomonas putida* mt-2, this pathway has been proposed to consist of two segments: an upper pathway for the transformation of furanic compounds into furoic acid (FA) and a common lower pathway for FA assimilation (Igeño et al. 2019). The *hmfABCDE* gene cluster was first identified in *Cupriavidus basilensis* HMF14, where it was characterised as being responsible for furoic acid metabolism (Fig. 1, lower pathway) (Koopman et al. 2010). Since then, it has been extensively studied in various bacterial strains. All species analysed for furfural assimilation possess this complete set of genes. However, the specific genes involved in the transformation of furanic compounds into FA (Fig. 1, upper pathway) remain largely unidentified in most bacteria. In *Ectopseudomonas oleovorans* CECT 5344 R1D, its growth on furfural follows an oxidative pathway regulated by AraC-family activators (Donoso et al. 2021; Igeño et al. 2019).

To gain a deeper understanding of the dynamics and kinetics of these biotransformation processes, real-time monitoring techniques are essential. While benchtop Nuclear Magnetic Resonance (NMR) spectroscopy has established itself as a robust tool for monitoring chemical synthesis (Silva Elipe 2025), its application to complex microbial processes, ranging from lipid accumulation to biocatalytic synthesis, remains a developing field (Bouillaud et al. 2020, 2019; Claaßen et al. 2020; Kreyenschulte et al. 2015; Legner et al. 2019; Mahler et al. 2025; Mehendale et al. 2020; Phuong et al. 2025). The inherent challenges of in-line monitoring, such as signal broadening due to sample turbidity and the overlap of complex extracellular metabolomes, often limit the resolution of transient intermediates in high-density or inhibitory cultures.

Such challenges are particularly relevant for 5-hydroxymethylfurfural (HMF) biotransformation, where substrate-induced inhibition and the transient formation of intermediates like BHMF pose significant analytical hurdles. In this work, we address these limitations by implementing a low-field in-line NMR setup to resolve the complex kinetics of aldehyde detoxification in the *E. oleovorans* CECT 5344 R1D enzymatic system. This monitoring is facilitated by the strain’s ability to excrete metabolites into the culture medium, a phenomenon previously documented in related bacteria (Lechtenberg et al. 2024), thereby allowing for straightforward extracellular analysis without the need for cell disruption.

By adopting a dual experimental regime, combining single-pulse batch kinetics to establish tolerance thresholds with multi-pulse fed-batch operations, we provide a comprehensive stoichiometric characterisation often missing in broader real-time monitoring studies. This approach specifically enables the observation of the strain’s dynamic metabolic shift: the initial excretion and subsequent re-uptake of biotransformation intermediates. Using this integrated strategy, we investigated HMF conversion kinetics, metabolic stability over 125 h, and the substrate scope of the bacterial aldehyde dehydrogenases against other environmentally relevant aldehydes.

## Materials and methods

### Chemicals

Ethanol, aldehydes, and all inorganic compounds were of analytical grade and used as received, without further purification. Rifampicin solution (40 mg/mL) was prepared in DMSO and sterilised by filtration.

A 100 mM stock solution of HMF was prepared by dissolving 1261 mg of HMF in distilled water. The solution was adjusted to pH 7 using 1 M NaOH, and the volume was brought up to 100 mL with distilled water. This stock solution was sterilised by autoclaving and subsequently diluted in the culture medium to the desired concentration for each HMF biotransformation experiment.

### Bacterial strains and culture conditions

Strain R1 was obtained as a spontaneous mutant of *Ectopseudomonas oleovorans* CECT 5344 resistant to rifampicin (40 μg/mL) (Quesada et al. 2007). The evolved strain R1D was obtained after four serial transfers of strain R1 to an M9 medium with 10 mM furfural as the sole carbon source. It was demonstrated that R1D was a punctual mutant in the *araC*-type regulator, capable to assimilate FA, and therefore furfural and furfuryl alcohol, efficiently (Igeño et al. 2019). In this study, strain R1D was used to perform the assays.

Luria-Bertani (LB) medium supplemented with rifampicin (40 μg/mL) was routinely used to grow the bacteria prior to the biotransformation experiments. M9 minimal medium (MM) (Maniatis et al. 1982) was used in the biotransformation experiments and was supplemented with a trace-element solution. Its composition was as follows: 10.75 g/L MgCl_2_, 6.16 g/L MgSO_4_·7 H_2_O, 4.75 g/L FeSO_4_·7 H_2_O, 2.0 g/L CaCO_3_, 1.44 g/L ZnSO_4_·7 H_2_O, 1.12 g/L MnSO_4_·7 H_2_O, 0.28 g/L CoSO_4_·7 H_2_O, 0.25 g/L CuSO_4_·5 H_2_O, 0.06 g/L H_3_BO_3_ and 51.3 mL/L HCl 12 N. NH_4_Cl was added at a final concentration of 15 mM as the sole nitrogen source and hydroxymethylfurfural (HMF) was added in the specified concentration as the sole carbon source. Both culture media were adjusted to pH 8.5 before use (Luque-Almagro et al. 2005).

Biotransformation assays were carried out using a 1:10 dilution of an overnight culture into fresh M9 minimal medium (~ 0.13 OD_600_; 10^8^ CFU/mL). Precultures were concentrated by centrifugation (13,000 × g, 2 min) and subsequent resuspension in one-tenth of the M9; this process was repeated twice before the precultures were used to inoculate the main experiment. Cultures were incubated in Erlenmeyer flasks filled to 1/10 of their nominal volume to ensure aerobic conditions and shaken at 200 rpm at 30 °C. Batch and fed-batch experiments were initiated by adding the appropriate volume of a stock HMF 100 mM solution to achieve the desired concentration in the culture. Bacterial growth was monitored by measuring the optical density at 600 nm (OD_600_).

### HMF toxicity assays

Bacteria were inoculated as described before, in M9 minimal medium containing HMF concentrations ranging from 10 to 300 mM. At regular intervals, 1 mL aliquots of the culture were withdrawn, centrifuged to remove cells, and the supernatants were frozen at −20 °C for further analysis in an 80 MHz benchtop NMR spectrometer. After 6 days, the number of viable cells (CFU/mL) was determined by serial dilution and plating on rifampicin LB agar (1.5% w/v bacto agar), followed by incubation at 30 °C to allow colony formation.

### Bacterial viability in mineral medium with and without HMF

A 1:10 dilution of the inoculum was cultured in M9 minimal medium both with and without 10 mM HMF to evaluate cell viability. Samples were withdrawn at the initial time point and after 24, 48 and 72 h. These were plated in LB agar (1.5% w/v bacto agar), supplemented with rifampicin at pH 8.5, and the CFU/mL were recorded. The experiment was performed in quadruplicate. The CFU/mL of the overnight LB cultures used for inoculation was determined to ensure that starting cell densities were consistent across all replicates.

### NMR instrumentation and acquisition parameters

^1^H NMR spectra were acquired using a Magritek Spinsolve 80 ULTRA benchtop spectrometer operating at a magnetic field strength of 1.88 T, corresponding to a ^1^H Larmor frequency of 80 MHz. To mitigate the dominant water signal in the culture medium, experiments were conducted using the Water Suppression Enhanced through T1 relaxation (WET) sequence. Data acquisition parameters were optimised for quantitative accuracy, including a spectral width of 2500 Hz, 65,536 data points, and the accumulation of 128 scans. A repetition time (T_R_) of 10 s was employed to ensure full longitudinal relaxation (T_R_ > 5 × T1), based on the relaxation times measured for HMF and its derivatives in the culture medium (T1 < 1.5 s). Spectra were processed using MestreNova software (Mestrelab Research S.L. 2019). A Gaussian apodisation function of 0.6 Hz was applied to free induction decay (FID) signals before Fourier transform, and automatic phase and baseline correction algorithms were employed.

### ^1^H NMR real-time biotransformation monitoring setup

The biotransformation was monitored using an in-line analytical configuration, where the culture medium was continuously circulated through a closed-loop bypass connected to the NMR spectrometer. This setup allowed for real-time kinetic data acquisition without manual sampling. The reaction monitoring system consisted of a peristaltic pump and a glass flow tube connected to the culture flask through 1 mm ID PTFE tubing. The flow tube featured an expanded 4.2 mm ID section at the measurement zone, positioned within the bore of the magnet. Prior to each experiment, the system was sterilised by circulating ethanol at 100 rpm for 10 min, followed by autoclaved distilled water for 1 h. Measurements were performed in stop-flow mode, with the pump operating at 20 rpm (approximately 12 mL/min), and spectra were acquired every 40 min. To maintain optimal magnetic field homogeneity and spectral resolution throughout long-term experiments, an automated shimming procedure was performed on-sample every 10 spectra.

### Quantitative NMR (qNMR) analysis and validation

Absolute concentrations of HMF and its metabolites, HMFCA and 2,5-bis(hydroxymethyl)furan (BHMF), were determined by integrating characteristic non-overlapping signals in the aromatic region (δ 6.4–7.6 ppm). Quantification was primarily performed through internal normalisation, using the initial HMF concentration (measured by weight and verified by the first NMR spectrum at *t* = 0) as the reference standard. This approach was intentionally chosen to avoid the addition of internal standards directly into the active culture, preventing potential metabolic interference or the introduction of unintended carbon sources. To further validate this method, independent experiments were conducted where representative culture aliquots were collected and centrifuged to remove the cells. Formic acid (10 mM) was then added to these cell-free supernatants as an internal standard. The consistency between the formic acid-referenced data and the internal normalisation method confirmed the accuracy of the concentration profiles and ensured precise chemical shift referencing (δ 8.44 ppm for the formate proton).

The linearity of the benchtop spectrometer for furanic compounds was previously validated (*R*^2^ > 0.999) within the 0.5–50 mM range. Integration boundaries were kept constant across each time-series to ensure reproducibility. In cell-free medium, the limit of quantification (LOQ) was determined to be 1 mM based on a Signal-to-Noise Ratio (SNR) > 10 for the HMF aromatic signals at 128 scans. However, the presence of the bacterial culture introduced a slight increase in background noise, raising the practical LOQ to 2 mM during the biotransformation experiments. Despite this, the sensitivity remained more than sufficient to accurately characterise the biotransformation kinetics and stoichiometric balances across the concentration ranges studied (10–50 mM). The stoichiometry of the process was verified by the constant sum of the relative integrals of the furanic ring protons, ensuring that no other major NMR-visible metabolites were formed.

## Results

### Identification of the products of HMF biotransformation

**Fig. 2:**
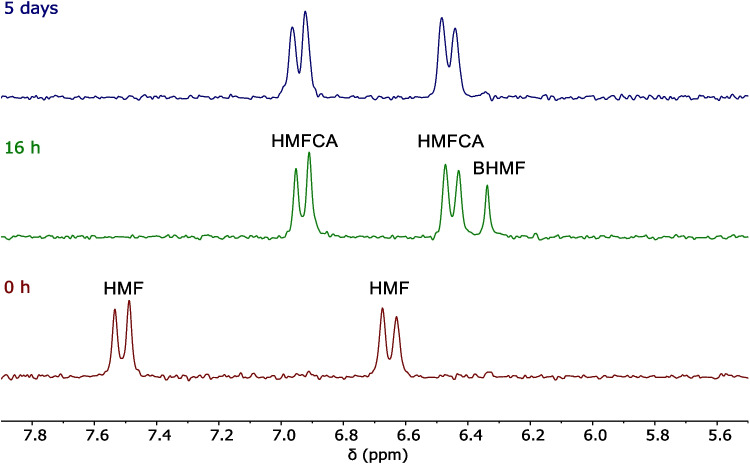
Biotransformation of HMF (10 mM) with *E. oleovorans* CECT 5344 R1D. The ^1^H NMR spectra correspond to aliquots withdrawn from the culture medium at the initial time (0 h), 16 h and 5 days. 5-Hydroxymethylfurfural (HMF) was completely biotransformed into 5-hydroxymethyl-2-furancarboxylic acid (HMFCA) and 2,5-bis(hydroxymethyl)furan (BHMF) in 16 h

*E. oleovorans* CECT 5344 R1D was inoculated into minimal medium containing 10 mM HMF as the sole carbon source and incubated as described in the M&M section. Complete consumption of HMF was observed within approximately 13 h, as determined by thin-layer chromatography (TLC) analysis of the culture medium. ^1^H NMR analysis confirmed the disappearance of the singlet at 9.46 ppm, corresponding to the formyl group of HMF, along with the two doublets at 7.54 and 6.68 ppm, attributed to its aromatic protons. Two new doublets at 6.96 and 6.48 ppm were assigned to the aromatic protons of HMFCA. This assignment was corroborated by comparison with an authentic standard (Fig. 2) and identified the major product previously designated as “unknown” in Igeño et al. (2019). A minor singlet at 6.36 ppm transiently appeared after 16 h incubation but subsequently decreased to undetectable levels after 5 days (Fig. 2). This signal was definitively assigned to BHMF based on reference spectra, thereby correcting its previous misidentification as FDCA (originally based on HPLC data) in our earlier study (Igeño et al. 2019). In a separate experiment, *E. oleovorans* CECT 5344 R1D was shown to slowly oxidise BHMF to HMFCA (Figure S1), confirming its role as a transient intermediate in the metabolic pathway.

The WET NMR protocol efficiently suppressed the water peak, enabling optimal resolution of the aldehyde and aromatic signals. Although the CH_2_ signals of HMF, HMFCA and BHMF were often obscured or overlapped with the residual water signal, compound identities were verified by comparison with authentic standards.

### Optimisation of the culture conditions

Culture pH and temperature were set according to previously established optima for *E. oleovorans* CECT 5344 R1D (Igeño et al. 2019). To determine the minimum biomass concentration required to overcome the inherent toxicity of HMF and enable its biotransformation, increasing dilutions of the inoculum were independently cultured with 10 mM HMF. A 1:10 dilution of the inoculum, corresponding to an approximate cell count of 10^8^ CFU/mL, was sufficient to completely transform HMF within approximately 13 h. In contrast, lower inoculum sizes did not achieve complete HMF conversion after 24 h. Based on these results, a 1:10 dilution of the inoculum (~ 0.13 OD_600_; 10^8^ CFU/mL) was used in all subsequent experiments.

**Fig. 3:**
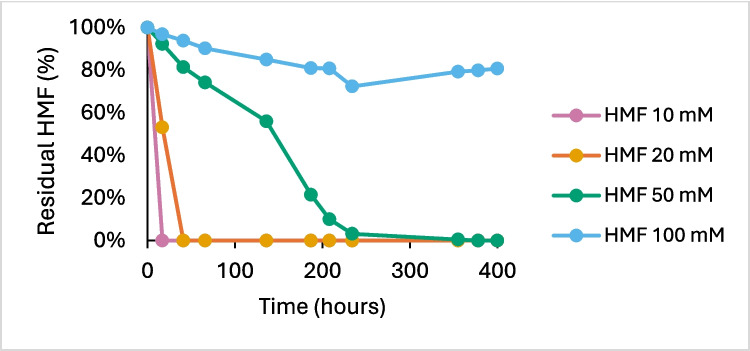
Biotransformation of different concentrations of HMF with *E. oleovorans* CECT 5344 R1D, quantified by ^1^H NMR. The highest biotransformation rate was observed at 10 mM of HMF. No transformation was observed in concentrations above 100 mM

Varying the initial HMF concentrations revealed tolerance up to 100 mM, though the biotransformation rate began to decline at much lower levels (Fig. 3). No transformation occurred at 200 or 300 mM, even after 3 weeks. The highest biotransformation rate was observed at an initial HMF concentration of 10 mM.

### Bacterial viability in mineral medium with and without HMF

Bacterial viability was monitored in M9 minimal medium with and without 10 mM HMF in the absence of any other assimilable carbon sources to isolate the effects of HMF and its derivatives. CFU/mL counts were determined at initial time, and after 24, 48 and 72 h. Although HMF was completely biotransformed within 24 h, the viability experiment was extended to 72 h to account for the potential effect of the slow oxidation of BHMF, which remained detectable in the medium for up to 5 days.

Across four independent experiments (Table S1 and Figure S16), cultures supplemented with HMF maintained higher viability during the first 24 h relative to controls. This trend did not reach statistical significance (Student’s *t*-test, *p* = 0.07).

### Real-time biotransformation monitoring by ^1^H NMR

Benchtop ^1^H NMR enabled real-time monitoring of the biotransformation and precise determination of the optimal harvesting time to maximise HMFCA yield. *E. oleovorans* CECT 5344 R1D cells were incubated in an M9 minimal medium containing 10 mM HMF and shaken at 200 rpm and 30 °C. The culture medium, containing the bacterial suspension, was circulated through the magnet bore of the NMR spectrometer in stop-flow mode. Spectra were acquired at 40-min intervals over a 16-h period.

**Fig. 4:**
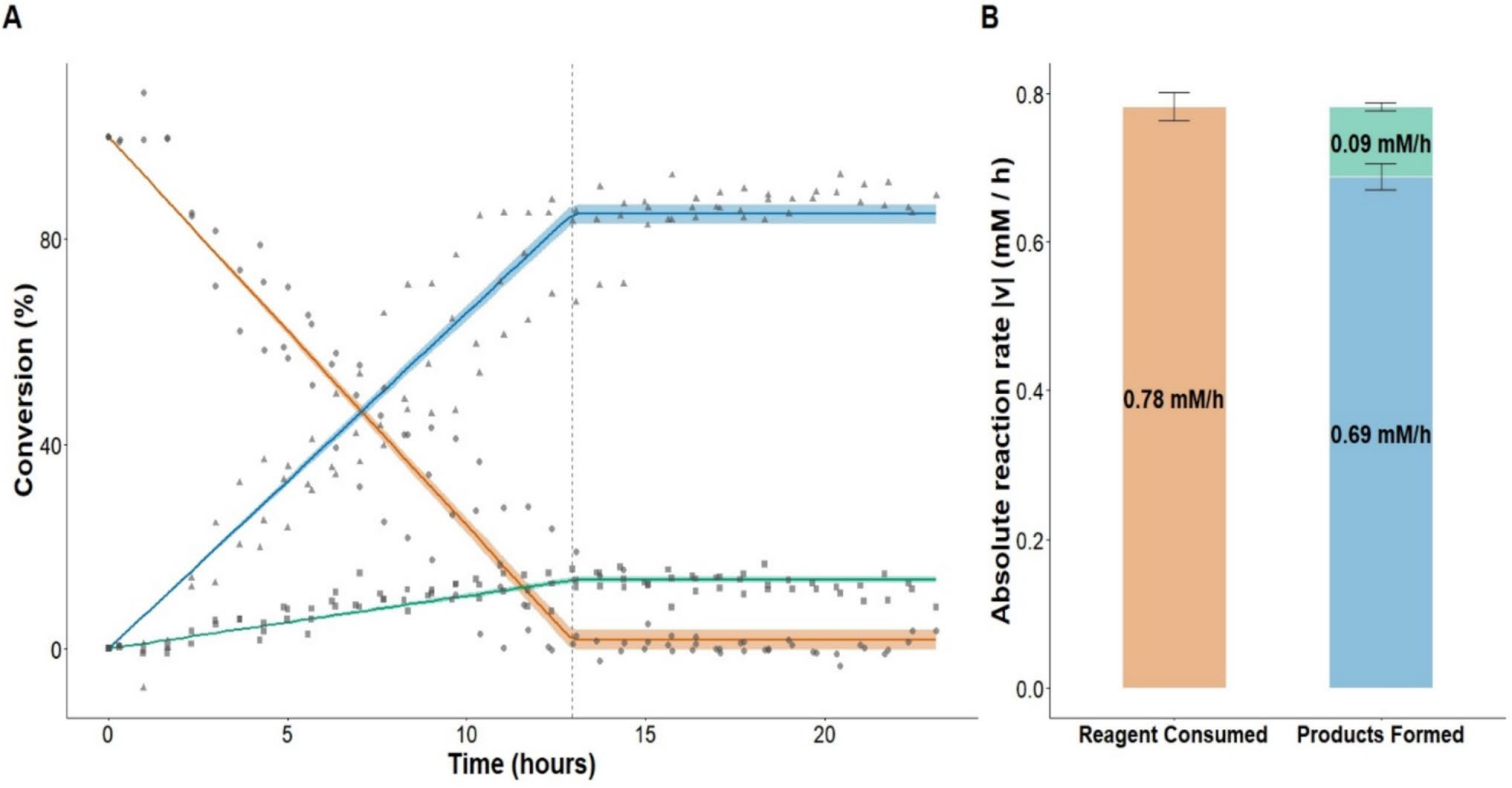
Kinetic profile and reaction rate balance for HMF biotransformation. **A** Concentration profiles of reaction compounds determined by NMR signal integration. Symbols represent experimental data from three independent replicates (brown circle (  ) HMF, blue triangle (  ) HMFCA, green square (  ) BHMF); solid lines indicate the segmented regression model with fixed initial conditions (100% for HMF; 0% for products). Shaded areas represent the 95% confidence intervals determined by bootstrapping (*n* = 1000). The vertical dashed line indicates the calculated HMF depletion time. **B** Mass balance of absolute zero-order reaction rates (v) for the primary consumption of HMF and the simultaneous formation of HMFCA and BHMF. Error bars correspond to the standard error of the kinetic rates (mM h^−1^)

The intensity of HMF signals decreased progressively, while new peaks corresponding to HMFCA and BHMF emerged in successive spectra. Complete HMF conversion was achieved within 13 h (Fig. 4A). Quantification of the different chemical species was obtained by integrating the relevant ^1^H NMR signals.

Mass balance of the absolute reaction rates showed that the cumulative rate of product formation (*v*_*HMFCA*_ + *v*_*BHMF*_) closely matched the HMF consumption rate (Fig. 4B). Following the exhaustion of HMF, a slower phase was observed, during which BHMF persisted and was only fully converted to HMFCA in extended experiments lasting several days (Fig. 2).

### Fed-batch operation

**Fig. 5:**
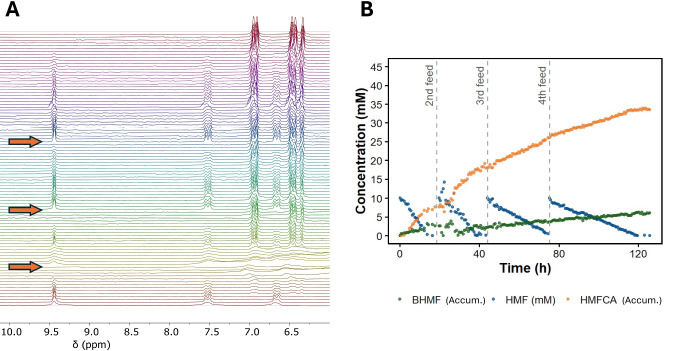
Real-time monitoring and stoichiometric analysis of HMF fed-batch biotransformation by *E. oleovorans* CECT 5344 R1D. **A** Stacked ^1^H NMR spectra (80 MHz) acquired in-line over 125 h, showing the consumption of HMF (blue) and the sequential formation of BHMF (green) and HMFCA (orange). **B** Cumulative molar concentration profiles derived from NMR integrals. Data were normalised within each feeding cycle to a 10 mM total furan load to compensate for solvent evaporation. Vertical dashed lines indicate the timing of successive 10 mM HMF additions (feeds 2 to 4). Q_P_ and yield values for each cycle are summarised in Table 2

To enhance the process productivity while mitigating the toxicity and reduced biotransformation rates associated with higher HMF concentrations, a fed-batch protocol with four successive HMF additions was implemented and monitored in real time by NMR (Fig. 5). Complete HMF conversion was observed after each addition, indicating sustained biotransformation capacity (Table 2).

**Table 2:** Volumetric productivities and molar yields for HMFCA and BHMF across successive feeding cycles as determined by in-line ^1^H NMR

Pulse	Q_p_HMFCA_^a^	HMFCA yield	BHMF yield	Total yield
P1	0.434	76.1	26.2	102.3
P2	0.424	103.1	−4.4	98.8
P3	0.259	80.4	14.5	94.9
P4	0.154	75.7	24.6	100.3

^a^*Q*_*P*_, volumetric productivity calculated as Δ[HMFCA]/Δt

### Biotransformation of other aldehydes

To assess substrate scope, cultures were incubated with a panel of aldehydes under the same culture conditions used for HMF, at final concentrations of 10, 5 and 2 mM. The aldehydes tested included: benzaldehyde, *p*-anisaldehyde, 4-hydroxybenzaldehyde, 4-dimethylaminobenzaldehyde, piperonal, salicylaldehyde, vanillin, syringaldehyde, 2-bromobenzaldehyde, 4-nitrobenzaldehyde, coniferaldehyde, 2-naphthaldehyde, fluorenal, *N*-methyl-2-pyrrolecarboxaldehyde, 2-imidazolecarboxaldehyde, *n*-heptaldehyde, crotonaldehyde and furfural (Table 3).

**Table 3:** Substrate scope and biotransformation efficiency of *E. oleovorans* CECT 5344 R1D towards various aldehydes

Substrate^a^	Initial conc. (mM)	Time (h)	Conversion (%)	Main product/outcome
Benzaldehyde	2	< 14	100	Benzoic acid
	10	< 14	100	Benzoic acid
Vanillin	2	< 17	100	Vanillic acid
	5	< 48	100	Vanillic acid
	10	67	47	Vanillic acid
Syringaldehyde	2	< 14	100	Syringic acid
	5	< 30	100	Syringic acid
	10	67	43	Syringic acid
Crotonaldehyde	2	< 14	100	Crotonic acid
*N*-Methyl-2-pyrrolecarboxaldehyde	2	< 14	100	*N*-Methyl-2-pyrrolecarboxylic acid
	5	48	62	*N*-Methyl-2-pyrrolecarboxylic acid
	10	72	40	*N*-Methyl-2-pyrrolecarboxylic acid
Furfural	2	< 14	100	Assimilation
	10	< 17	100	Assimilation

^a^No transformation was observed for 2 mM *p*-anisaldehyde, 4-hydoxybenzaldehyde, 4-dimethyaminobenzaldehyde, piperonal, salicylaldehyde, 2-bromobenzaldehyde, 4-nitrobenzaldehyde, coniferaldehyde, 2-naphthaldehyde, fluorenal, 2-imidazolecarboxaldehyde and *n*-heptaldehyde

As reported previously (Igeño et al. 2019), furfural was efficiently assimilated by the bacteria. ^1^H NMR analysis of the culture medium revealed the transient formation of furoic acid and furfuryl alcohol (Figures S2–S3) and a control experiment confirmed rapid metabolism of furfuryl alcohol (Figure S4). Significant transformation to the corresponding carboxylic acids was observed for benzaldehyde (Fig. 6 and Figure S5), vanillin, syringaldehyde, crotonaldehyde and *N*-methyl-2-pyrrolecarboxaldehyde (Figures S6–S15). No transformation was detected for the remaining aldehydes tested.

**Fig. 6:**
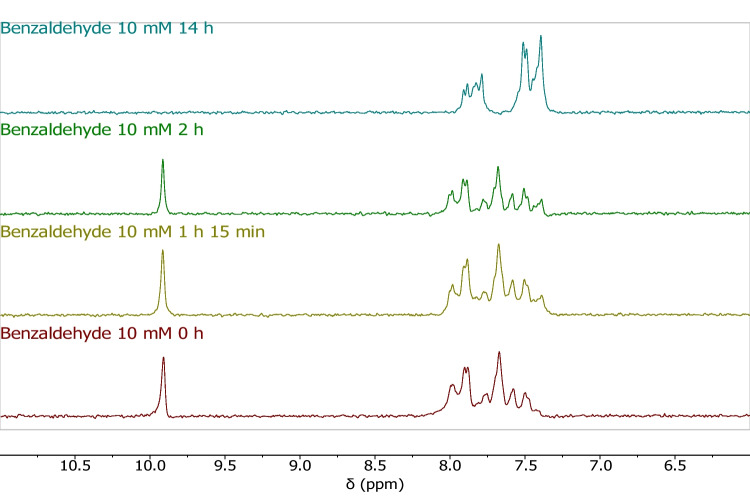
Stacked ^1^H NMR spectra for the biotransformation of benzaldehyde 10 mM into benzoic acid at different times. *E. oleovorans* CECT 5344 R1D completely transformed the aldehyde in approximately 14 h

## Discussion

Our results demonstrate that *E. oleovorans* CECT 5344 R1D rapidly converts HMF to HMFCA with transient formation of BHMF, which is subsequently oxidised to HMFCA. Together with prior observations in this strain (Igeño et al. 2019) and other bacteria (Liu et al. 2024; Xu et al. 2020; Zhang et al. 2017), these findings support a combined reductive/oxidative route mediated by non-specific alcohol and aldehyde dehydrogenases. Under the conditions tested, direct oxidation of HMF to HMFCA appears to be the dominant metabolic flux, whereas BHMF formation represents a minor, kinetically subordinate branch that is later re-oxidised. 

The kinetic profiles obtained by real-time ^1^H NMR with 10 mM HMF revealed an apparent biphasic behaviour. During the first phase, HMF decreased linearly until complete depletion, stoichiometrically mirrored by the formation of HMFCA and BHMF (Fig. 4A). This profile is consistent with zero-order kinetics within the tested concentration range, potentially suggesting that the enzymatic system operates near saturation ([HMF] >  > K_m_). Under these specific conditions, the reaction rate (*v*) appeared independent of substrate concentration, approximating a maximum catalytic potential (V_max_) for the given bacterial density. The robustness of this observation was supported by the mass balance of the absolute reaction rates (Fig. 4B), where the cumulative rate of product formation (*v*_*HMFCA*_ + *v*_*BHMF*_) closely matches the HMF consumption rate. This stoichiometric consistency, combined with the 95% confidence intervals derived from bootstrapping, suggests an efficient biotransformation process under the studied parameters. Furthermore, the transition to a second, slower kinetic phase after HMF exhaustion may point to a sequential reaction mechanism, likely involving the secondary transformation of BHMF, which only becomes apparent once the primary substrate is no longer available. However, this second phase exhibited a pseudo-stationary state with negligible slopes for the HMFCA and BHMF concentrations within the experimental timescale.

Importantly, increasing the initial HMF concentration above the optimal range did not improve catalytic performance. Although cells tolerated concentrations up to 100 mM, transformation rates declined and no conversion was observed at 200–300 mM. These findings are consistent with substrate inhibition/toxicity limiting flux through the aldehyde oxidation pathway (Lopachin and Gavin 2014). Consequently, a fed-batch strategy was implemented to maintain HMF within a sub-inhibitory window, sustaining productivity over multiple cycles and avoiding substrate overload.

In addition to concentration limits, operational parameters strongly influenced the efficiency of HMF biotransformation. The optimal ranges previously reported for *E. oleovorans* CECT 5344 R1D (Igeño et al. 2019) were adopted here, likely contributing to the robustness and reproducibility of HMF conversion. Given that aldehyde dehydrogenases and related oxidoreductases often display narrow activity optima, maintaining these parameters is essential for maximising catalytic flux through the oxidative pathway. Inoculum size also proved critical; we observed a sharp contrast between the complete HMF conversion at the selected cell densities (~ 10^8^ CFU/mL) and the lack of detectable activity at lower biomass loads. This observation suggests a minimum biological threshold required to overcome the inherent toxicity of furan aldehydes and initiate the oxidative pathway. The choice of biomass concentration was strategically balanced to satisfy both biological and analytical requirements. While higher cell densities are typically employed in industrial settings to maximise volumetric productivity, the current density was selected to prevent signal saturation and excessive viscosity within the NMR flow cell. Under the tested conditions, the kinetics followed an apparent zero-order model at 10 mM HMF, where enzymatic turnover becomes primarily limited by the total active enzyme pool provided by the cells.

Regarding cellular physiology, viability assays in the absence of other assimilable carbon sources revealed a reproducible tendency toward better survival in the presence of HMF during the first 24 h. Although the decrease in bacterial counts was notably less pronounced in HMF-supplemented cultures compared to the control, this trend did not reach formal statistical significance (Student’s t-test, *p* = 0.07), likely due to the inherent variability associated with CFU/mL measurements in starvation assays.

While this observation requires further confirmation, the potential for furan aldehydes to support bacterial viability is not without precedent. Although the detoxification of HMF typically imposes an energetic cost (Ask et al. 2013; Lopachin and Gavin 2014), certain bacteria can derive a net energetic benefit from this pathway. This has been demonstrated in strains such as *Cupriavidus basilensis* HMF14, which utilises HMF as a sole carbon and energy source (Koopman et al. 2010), and engineered *Pseudomonas putida* strains capable of assimilating furans for growth (Guarnieri et al. 2017). In our system, the metabolic benefit appears to stem from the oxidative conversion of HMF and BHMF into HMFCA. This oxidation likely generates the reducing power necessary for cellular maintenance under starvation conditions.

However, it is important to note that while HMF presence sustained viability, it did not support detectable biomass accumulation. Since HMFCA is not further metabolised and is efficiently excreted, it cannot contribute as an assimilable carbon source for growth. Therefore, in *E. oleovorans* CECT 5344 R1D, this pathway functions as a specialised detoxification mechanism that yields transient metabolic energy rather than a complete assimilative route for furan degradation.

This “metabolic dead-end” hypothesis is strongly supported by our ^1^H NMR monitoring. Assays using purified BHMF (5 mM) confirmed its slow but steady conversion into HMFCA, which then remained stoichiometrically stable for the remainder of the experiment (up to 144 h) with no further signal decay. The persistence of HMFCA, coupled with the absence of biomass accumulation during HMF or BHMF depletion, provides strong evidence that these furanic derivatives are not assimilated into central metabolism under the tested conditions. Instead, the persistent concentration of HMFCA suggests it acts as a metabolic end-product, effectively sequestering the carbon initially provided by HMF.

From an analytical standpoint, benchtop ^1^H NMR offers a robust alternative to chromatography-based methods, such as HPLC or LC-MS, which typically require discrete sampling, complex sample preparation (e.g., filtration, protein precipitation), and often derivatisation of polar intermediates. Our in-line NMR setup facilitates continuous, minimally invasive monitoring of the intact bioreactor broth. Unlike optical methods like Raman spectroscopy, which are frequently prone to fluorescence interference and signal scattering in turbid microbial cultures, the NMR workflow provides high-resolution profiling of both HMFCA and the transient intermediate BHMF without matrix-induced baseline distortion. While raw lignocellulosic hydrolysates would undoubtedly introduce greater spectral complexity, the ability of NMR, when coupled with appropriate suppression techniques, to resolve individual signals in complex mixtures offers a significant advantage for metabolic tracking.

The clarity of the spectra was strategically enhanced by the use of a defined mineral medium, which significantly simplified the metabolic background. While it has been reported that fed-batch production of HMFCA with supplemental carbon sources can improve bacterial viability, performing experiments without additional assimilable carbon, using only a mineral medium with HMF, offers distinct advantages. This approach ensures high product selectivity and prevents the accumulation of metabolic by-products that might otherwise interfere with the HMFCA signals in the NMR spectrum. This simplified environment was instrumental for the precise quantification of metabolic flux and the validation of the NMR-monitoring setup, providing a necessary baseline for future studies involving complex industrial feedstocks.

Although NMR is, in principle, capable of detecting both intra- and extra-cellular metabolites, the contribution of intracellular components was negligible due to the low effective intracellular volume and restricted molecular mobility within the crowded cytosolic environment (Bernadó et al. 2004; Wang et al. 2011). This biological stability, coupled with sustained cell viability, prevented the leakage of intracellular contents and ensured high signal-to-noise ratios. By intentionally designing the process to minimise analytical interference, we established a streamlined monitoring workflow that is directly transferable to other aldehyde detoxification processes where real-time kinetic data is critical for process control.

The substrate scope analysis highlights the ecological relevance and biocatalytic versatility of *E. oleovorans* CECT 5344 R1D against a variety of industrially and environmentally relevant aldehydes. The efficient oxidation of lignin-derived side-products, such as furfural, vanillin and syringaldehyde, is particularly noteworthy, as these compounds are known inhibitors of yeast fermentation in bioethanol production (Cao et al. 2014; Jayakody and Jin 2021). Furfural, a widespread environmental toxicant with documented cytotoxicity (Allen et al. 2010) and potential co-carcinogenic activity (Api et al. 2024; National Toxicology Program 1990), was rapidly biotransformed, likely through the same dehydrogenase machinery involved in HMF detoxification. This enzymatic breadth extends to structurally related heterocycles like *N*-methyl-2-pyrrolecarboxaldehyde, as well as other aromatic aldehydes such as benzaldehyde (Brühne and Wright 2011). Furthermore, the conversion of the highly reactive aliphatic toxicant crotonaldehyde (Garcia and Harbison 2015; Hampton et al. 1982; Sleiman et al. 2016; van Andel et al. 2006), noted for its cytotoxic and immunosuppressive effects in human cells (IARC Working Group on the Identification of Carcinogenic Hazards to Humans 2021; Liu et al. 2010), further illustrates the broad aldehyde-oxidation capacity of this strain. These results underscore the ecological and bioprocess potential of *E. oleovorans* CECT 5344 R1D for mitigating the toxic and inhibitory effects of diverse carbonyl compounds in complex industrial feedstocks.

Altogether, these biotransformations suggest that *E. oleovorans* CECT 5344 R1D possesses enzymatic systems adapted to detoxify a wide range of aromatic and unsaturated aldehydes commonly encountered in plant-associated and soil environments. This metabolic plasticity, characteristic of the *Pseudomonas* genus, enables the strain to thrive in diverse and often challenging ecological niches. Such adaptability has clear biotechnological value, revealing promising opportunities for aldehyde detoxification and valorisation in complex lignocellulosic feedstocks.

## Conclusion

This study shows that *Ectopseudomonas oleovorans* CECT 5344 R1D is a highly efficient biocatalyst for the sustainable production of HMFCA. While this strain had not been previously studied in depth for its ability to biotransform furanic aldehydes, it proved exceptionally effective for HMFCA accumulation, a trait that remains relatively uncommon in the literature.

Compared to other bacteria in the same family, such as *P. putid*a KT2440 (Xu et al. 2020) or *P. rhodesiae* NL2019 (Liu et al. 2023), our strain offers a distinct advantage in terms of efficiency. While those strains can achieve higher volumetric productivities, they require cell densities up to 100 times higher than those used in this work. In contrast, *E. oleovorans* CECT 5344 R1D achieves a quantitative molar yield with a much lower biomass load and, unlike some other microbes such as *P. putida* ALS1267 (Lee et al. 2016), it does not consume the HMFCA it produces. This establishes the strain as a remarkably clean and specific “cell factory” for transforming lignocellulosic inhibitors into high-value building blocks.

A key contribution of this work was the successful implementation of an in-line benchtop NMR loop to monitor the reaction in real time. By optimising the flow parameters and using a defined mineral medium to establish an analytical baseline, we overcame the challenges of maintaining a viable bacterial culture while ensuring high spectral resolution. This minimally invasive workflow was sensitive enough to identify the transient intermediate BHMF and precisely track its conversion into HMFCA, providing kinetic details that are usually lost with offline sampling.

Beyond HMF, the ability of strain CECT 5344 R1D to metabolise a wide range of other toxic aldehydes suggests its potential as a versatile tool for the pre-treatment of lignocellulosic hydrolysates, rendering them suitable for subsequent fermentation steps, such as bioethanol production. Overall, this work validates a powerful combination: a robust, non-consuming biocatalyst and a sophisticated, automated monitoring platform. The next steps, such as scaling up the process or implementing in situ product removal, will help move this technology towards practical use in sustainable, bio-based chemical production.

## Supplementary information

Below is the link to the electronic supplementary material.

ESM 1 (PDF 1.76 MB)

## Data Availability

All relevant data are within the manuscript and in the supporting information (SI).
