# Supplementary material for: Real-time NMR monitoring of HMF biotransformation by Ectopseudomonas oleovorans CECT 5344 R1D
Source: Appl Microbiol Biotechnol. 2026 May 7;110:196. doi: 10.1007/s00253-026-13853-7 (PMC13320085; doi:10.1007/s00253-026-13853-7)
Supplement: Supplementary file 1 [file 253_2026_13853_MOESM1_ESM.pdf]

Supplementary Information

Real-Time NMR Monitoring of HMF Biotransformation by *Ectopseudomonas oleovorans*

Mar Gómez-Ortega[a], Sara Diego[a,b], Felipe Morales-Durán[a], Faustino Merchán[a,*], Rafael Blasco[a], Ana G. Neo[b] and Carlos F. Marcos[b,*]

[a] Department of Biochemistry, School of Veterinary Sciences, Universidad de Extremadura, 10003 Cáceres, Spain.

[b] Laboratory of Bioorganic Chemistry & Membrane Biophysics (L.O.B.O.). Departamento de Química Orgánica e Inorgánica. Universidad de Extremadura. 10003 Cáceres, Spain.

Contents

1. Bacterial genes related to furan metabolism 1
2. ^{1}H NMR of aldehyde transformations 2
3. Bacterial viability with and without HMF 10

1. Bacterial genes related to furan metabolism

Table S 1. Genetic markers in *E. oleovorans* CECT 5344 R1D associated with furan metabolism and identity comparison with reference strains.

Protein (Gene ID)	Putative Function	*P. putida* ALS1267[a]	*P. putida* Fu1[b]	*C. basilensis* HMF14[c]
HmfA (BN5_2298)	Furoyl-CoA dehydrogenase (L)	89%	-	64%
HmfB (BN5_2299)	Furoyl-CoA dehydrogenase (FAD)	89%	-	59%
HmfC (BN5_2300)	Furoyl-CoA dehydrogenase (Fe-S)	90%	-	77%
HmfD (BN5_2301)	Furoyl-CoA synthetase	88%	-	61%
HmfE (BN5_2302)	2-Oxoglutaroyl-CoA hydrolase	90%	-	80%
BenE1 (BN5_2303)	Putative transporter (HmfI-like)	-	-	-
Hydrolase	Furan ring opening (hypothesized)	25%	24%	-
PsfD (BN5_2306)	Accessory factor	83%	84%	-
AraC (BN5_2307)	Transcriptional regulator	73%	73%	-

[a] Identity to *P. putida* ALS1267 (Crigler et al. 2020). [b] Identity to *P. putida* Fu1 (Nichols et al. 2012). [c] Identity to *C. basilensis* HMF14 (Koopman et al. 2010). Interestingly, key genes such as hmfI, hmfF, hmfT, psfG, psfA, psfB, and psfC (hmfG) were absent in the R1D genome, which correlates with the observed lack of ring-opening activity.

2. ^{1}H NMR of aldehyde transformations

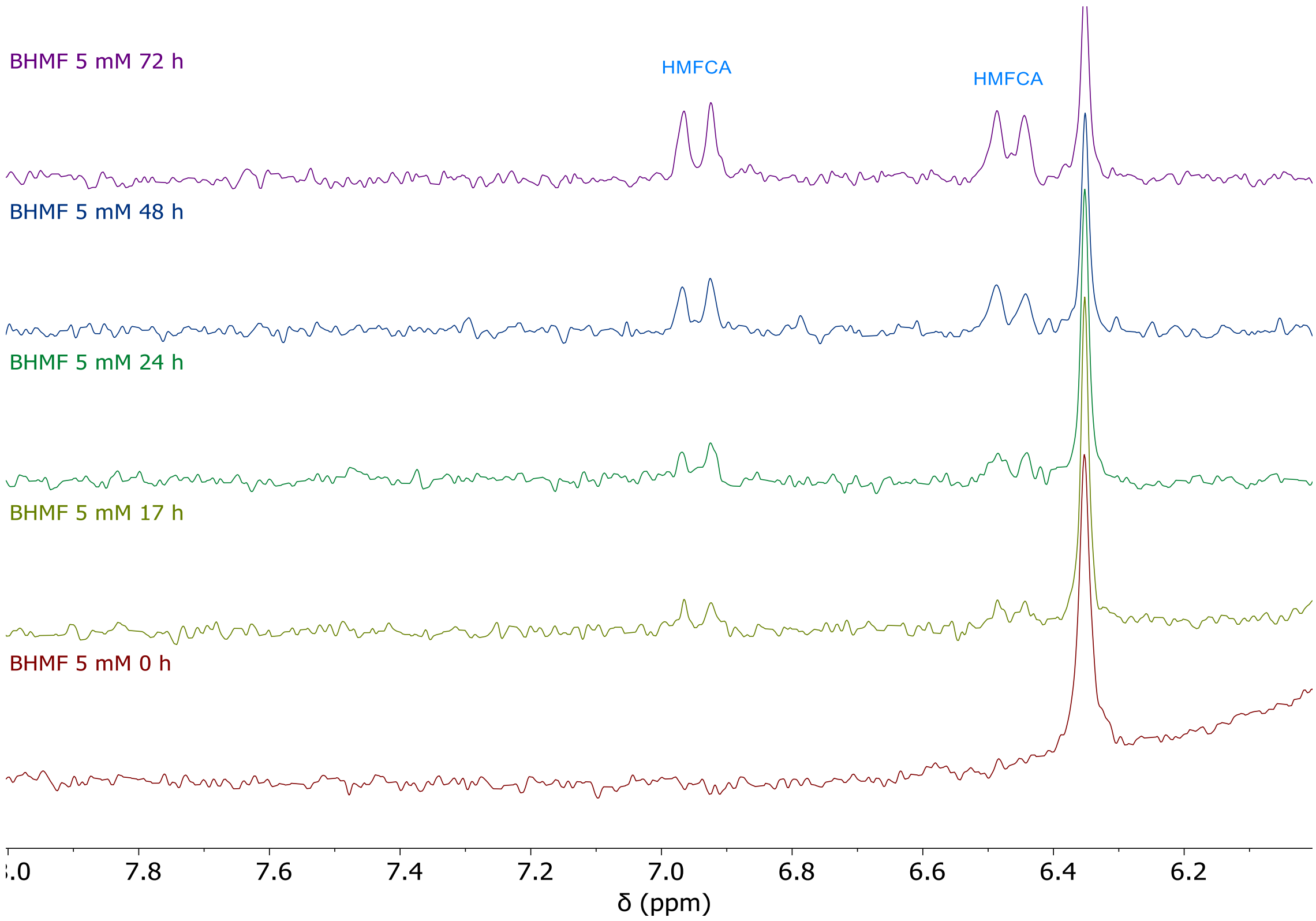

Figure S 1. Stacked ^{1}H NMR spectra for the *E. oleovorans* CECT 5344 biotransformation of 2,5-bis(hydroxymethyl)furan (BHMF) 5 mM at different times.

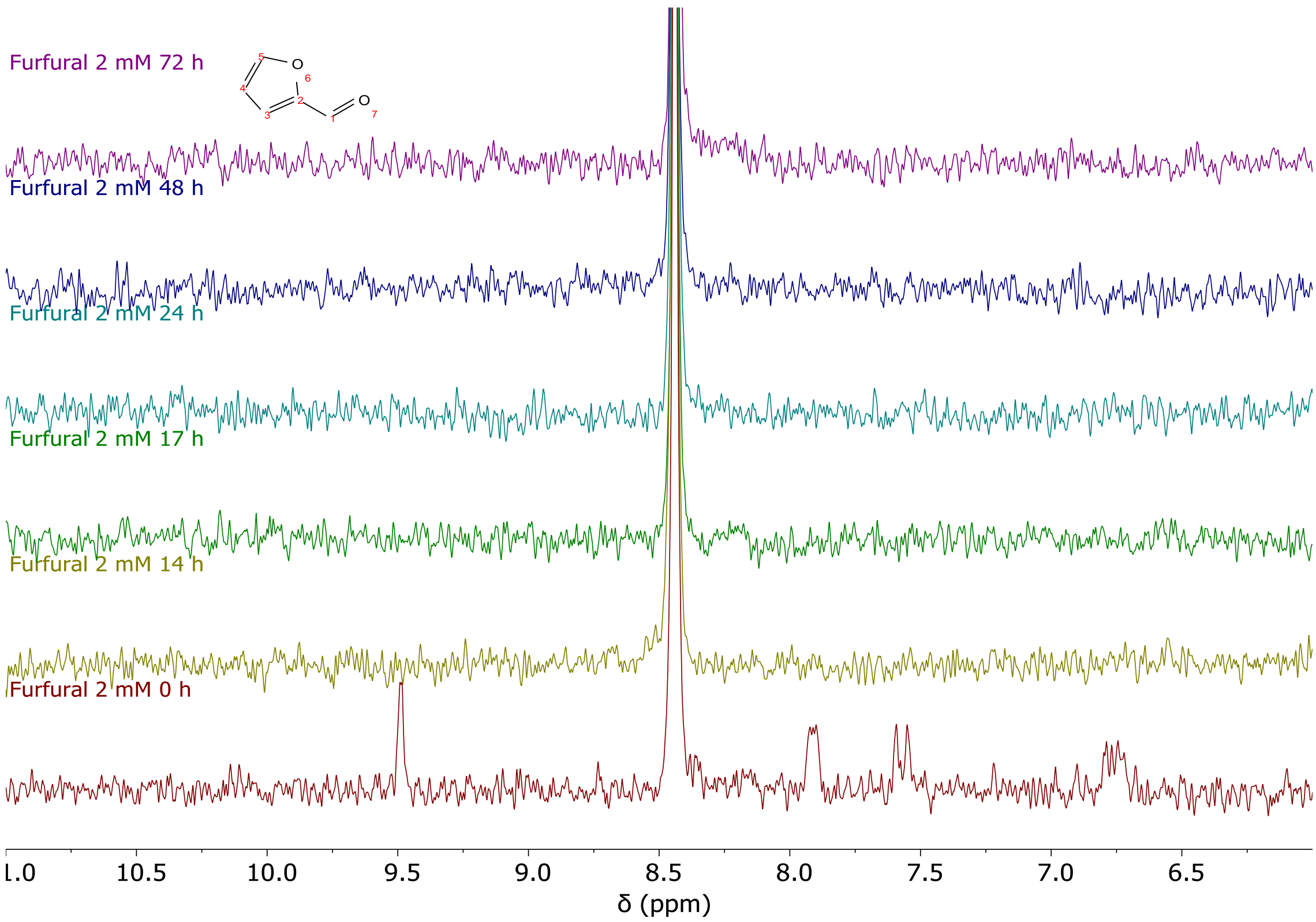

Figure S 2. Stacked ^{1}H NMR spectra for the *E. oleovorans* CECT 5344 biotransformation of furfural 2 mM at different times.

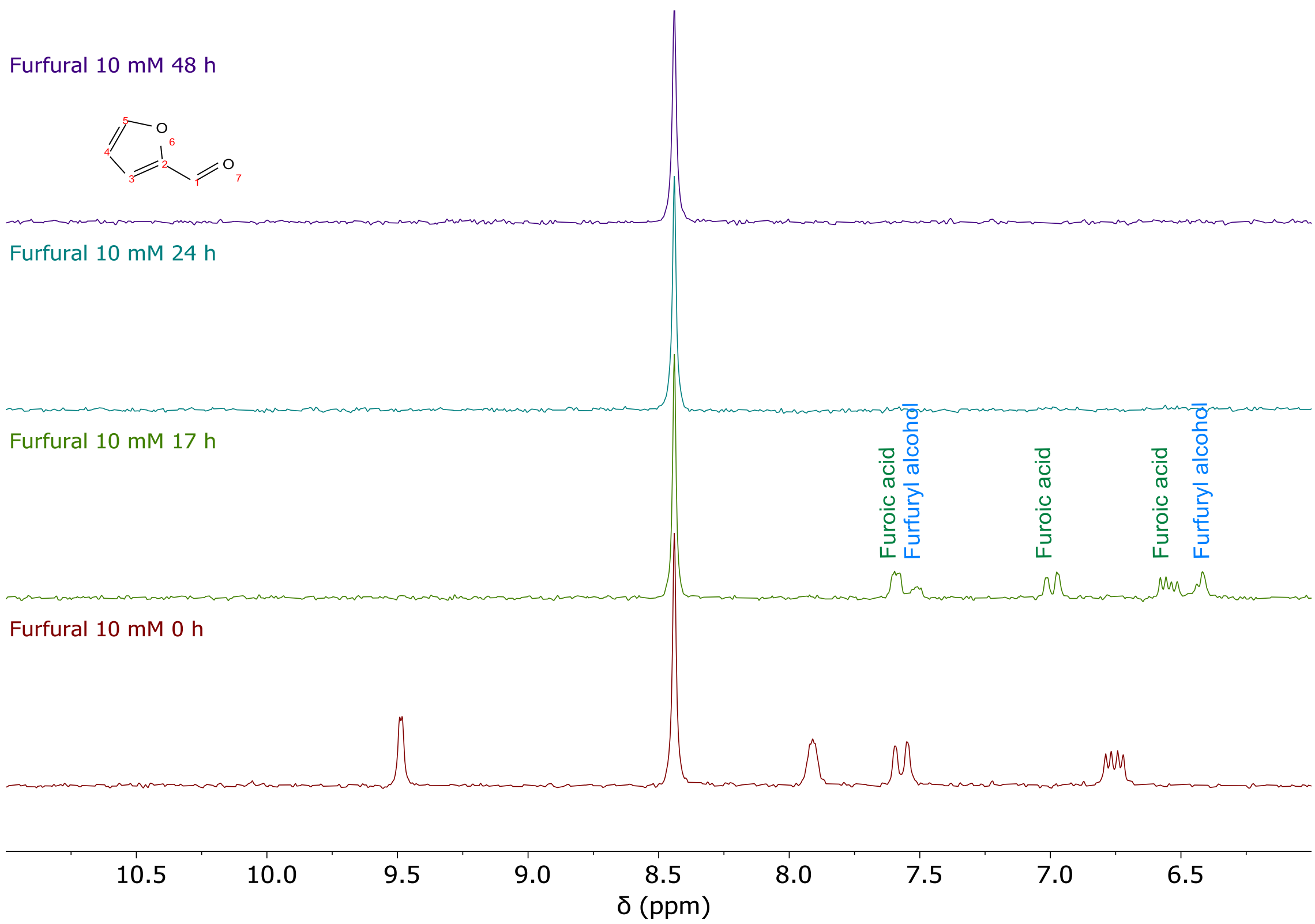

Figure S 3. Stacked ^{1}H NMR spectra for the *E. oleovorans* CECT 5344 biotransformation of furfural 10 mM at different times.

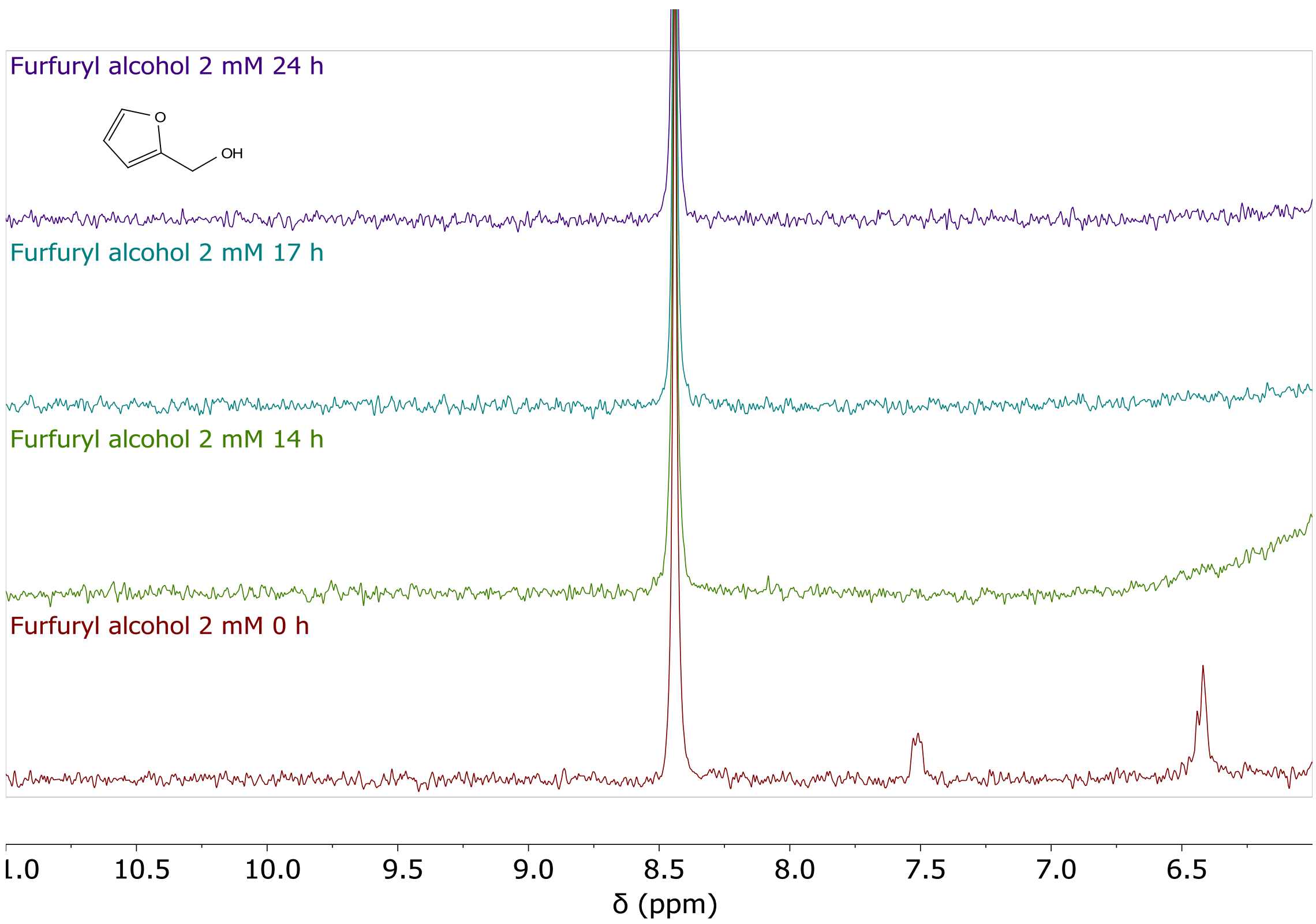

Figure S 4. Stacked ^{1}H NMR spectra for the *E. oleovorans* CECT 5344 biotransformation of furfuryl alcohol 2 mM at different times.

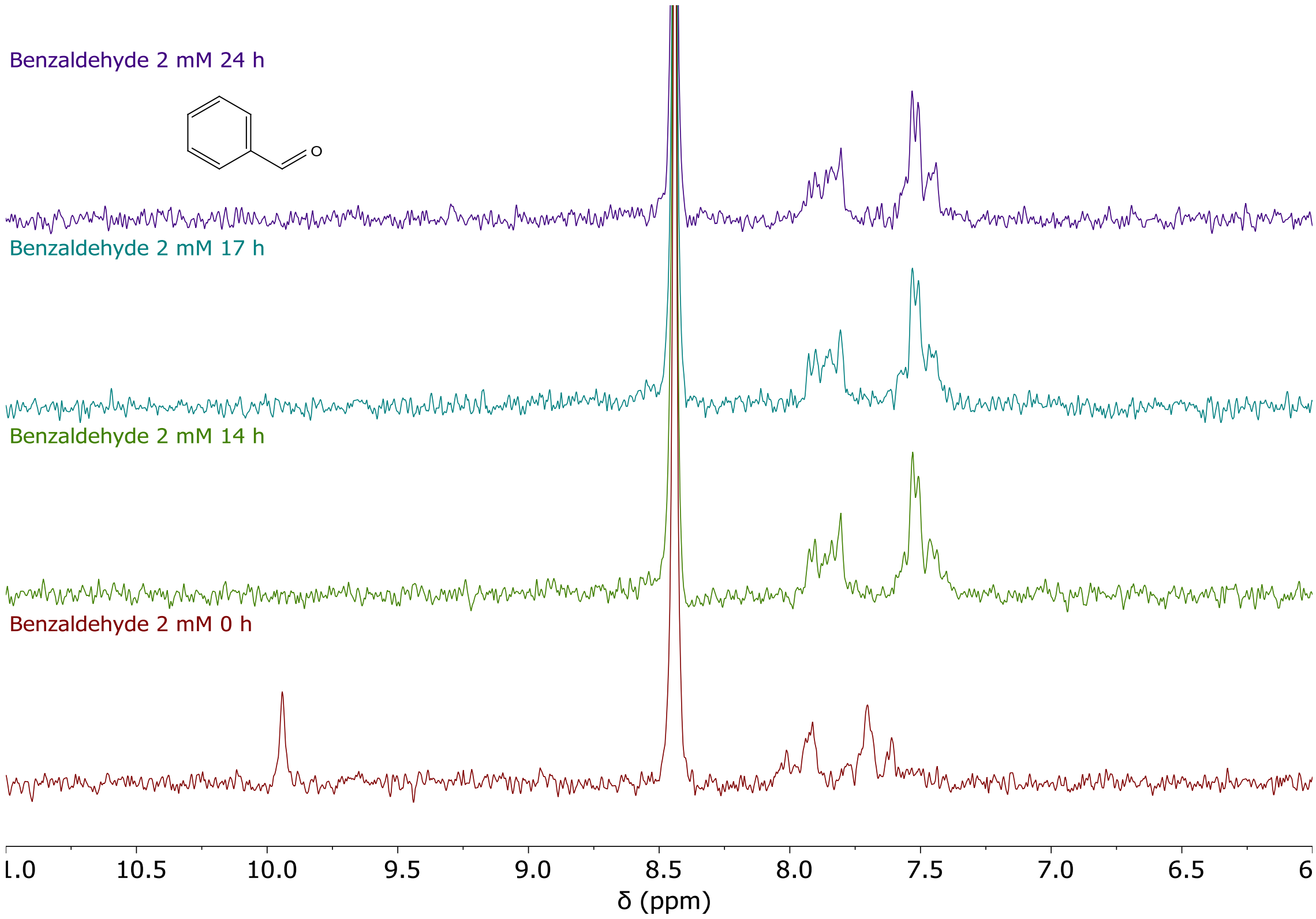

Figure S 5. Stacked ^{1}H NMR spectra for the *E. oleovorans* CECT 5344 biotransformation of benzaldehyde 2 mM into benzoic acid at different times.

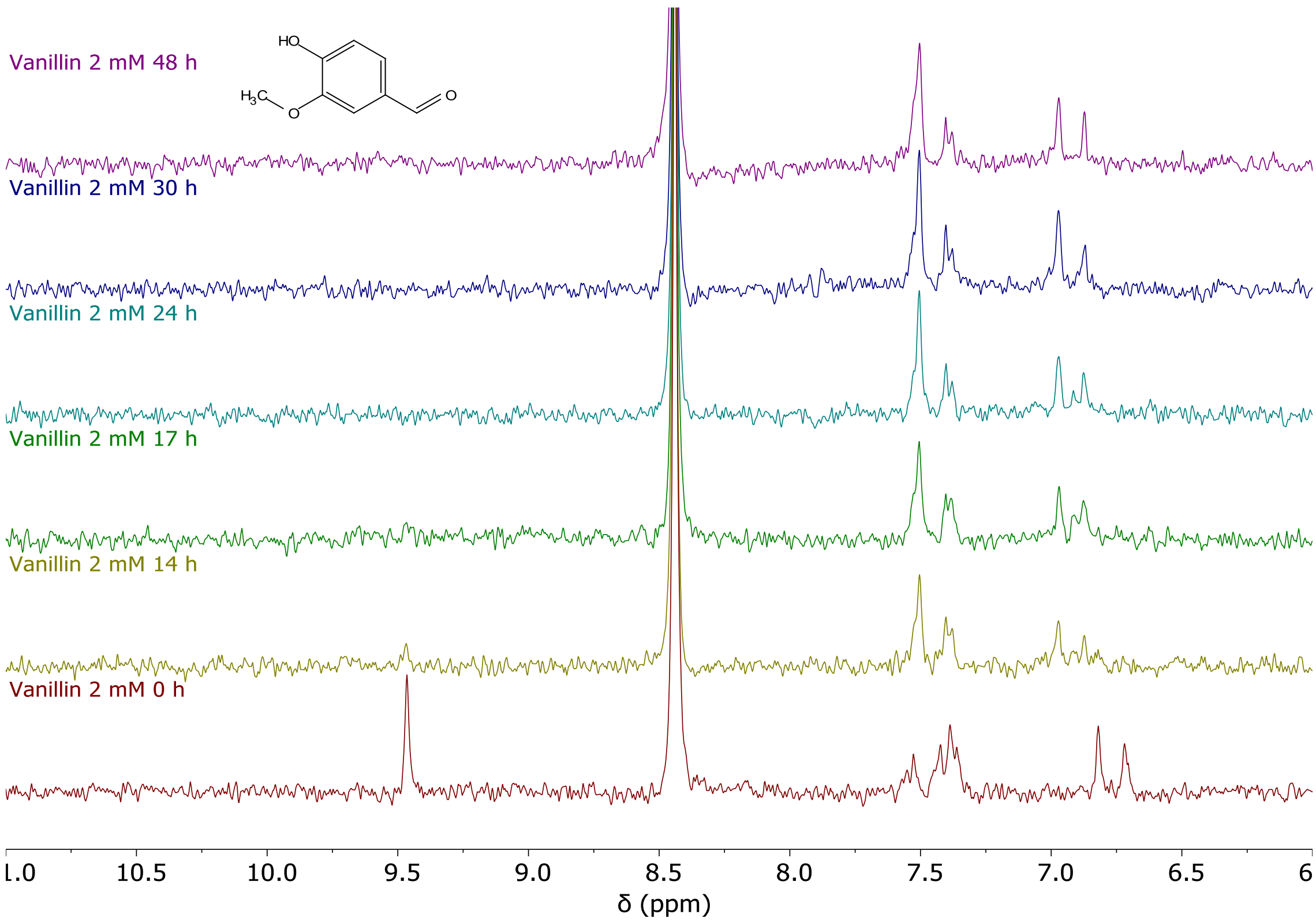

Figure S 6. Stacked ^{1}H NMR spectra for the *E. oleovorans* CECT 5344 biotransformation of vanillin 2 mM at different times.

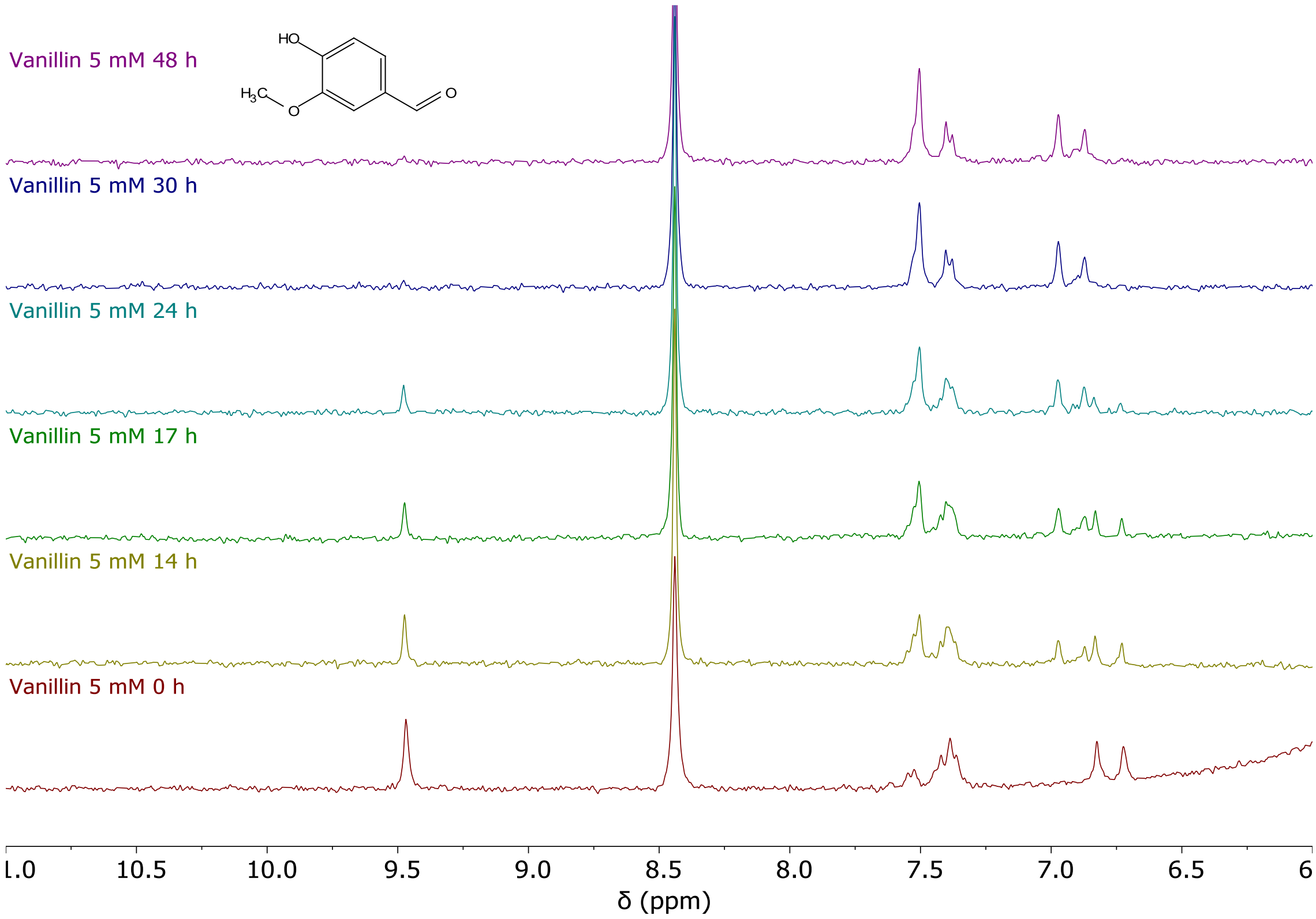

Figure S 7. Stacked ^{1}H NMR spectra for the *E. oleovorans* CECT 5344 biotransformation of vanillin 5 mM at different times.

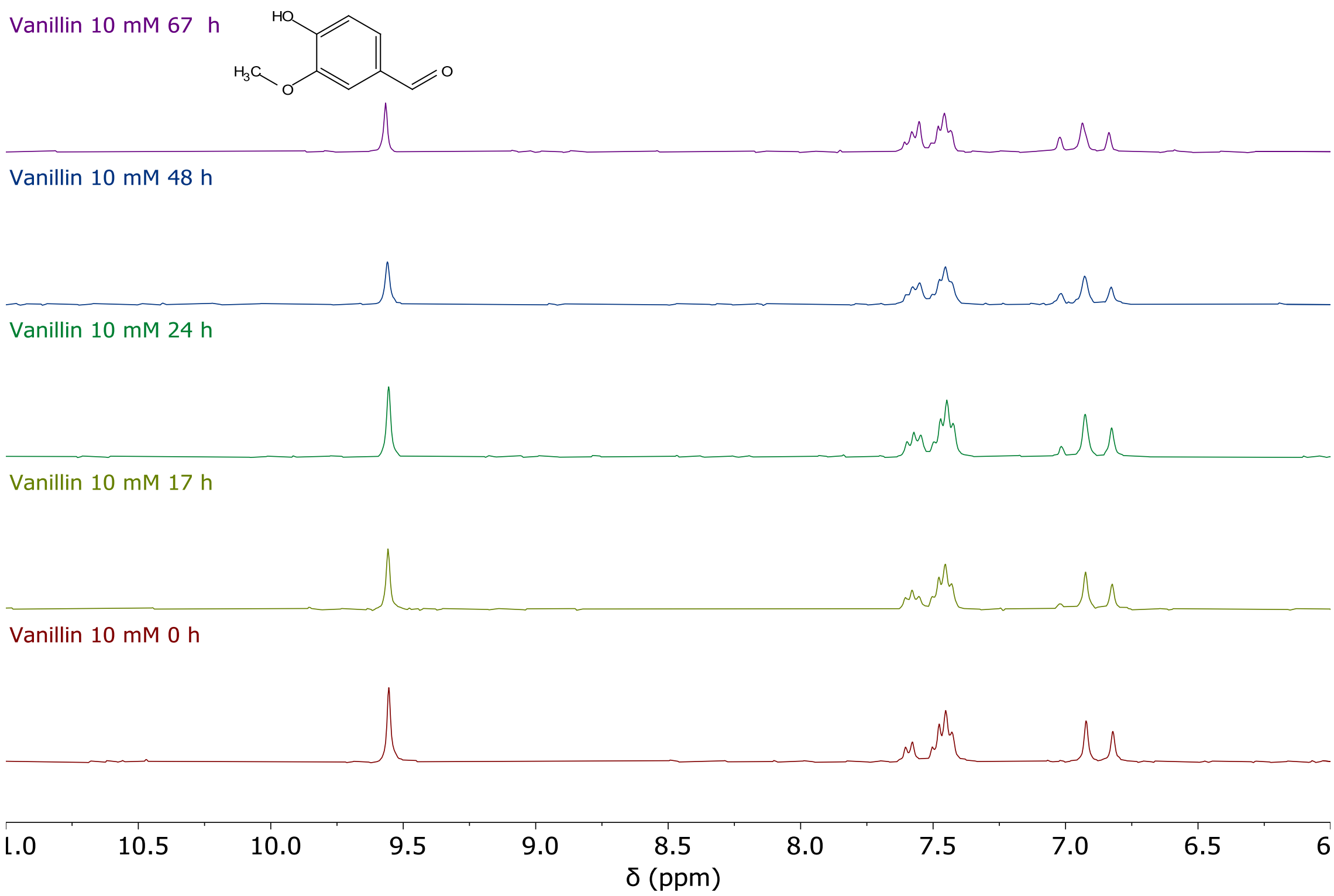

Figure S 8. Stacked ^{1}H NMR spectra for the *E. oleovorans* CECT 5344 biotransformation of vanillin 10 mM at different times.

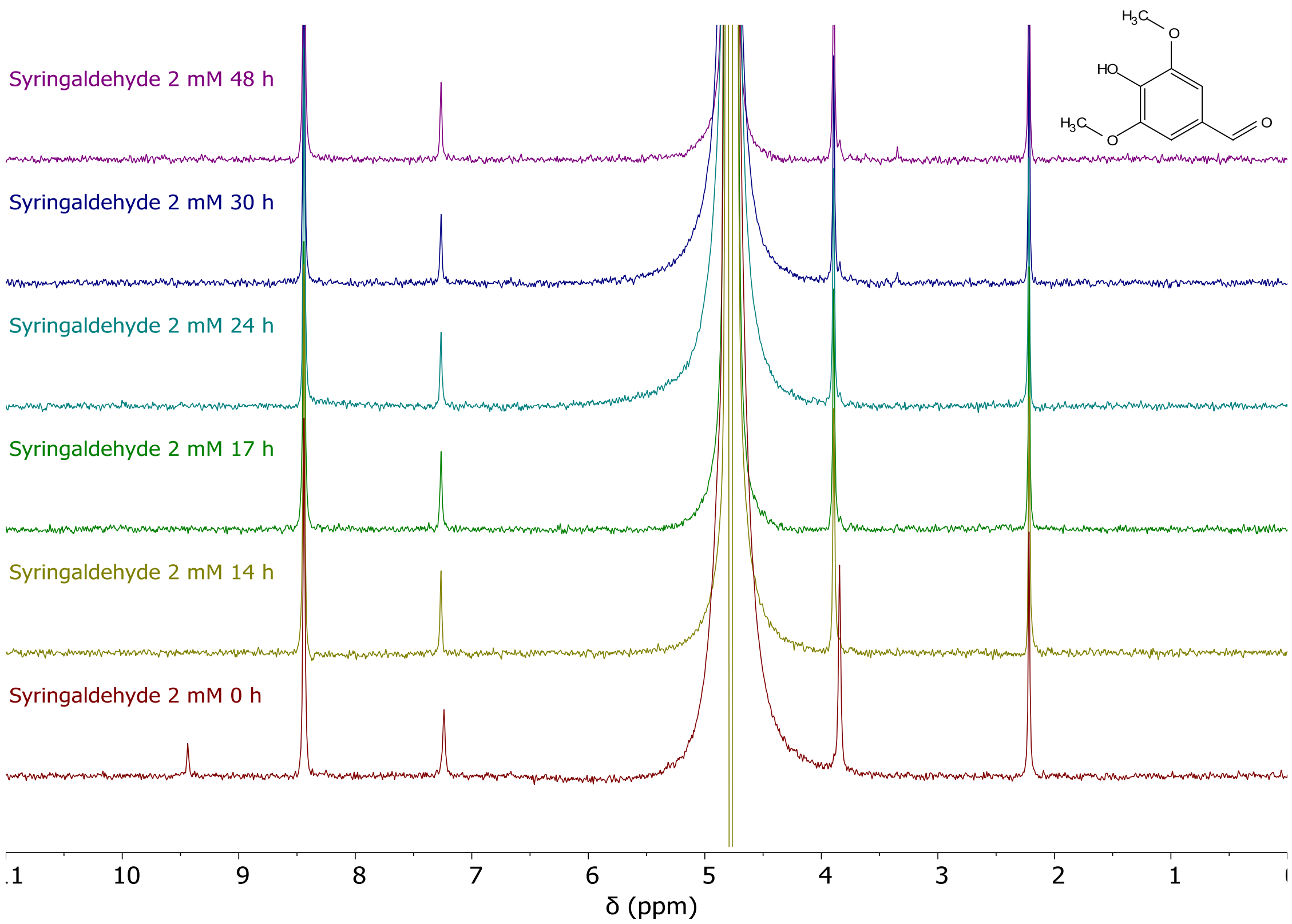

Figure S 9. Stacked ^{1}H NMR spectra for the *E. oleovorans* CECT 5344 biotransformation of syringaldehyde 2 mM at different times.

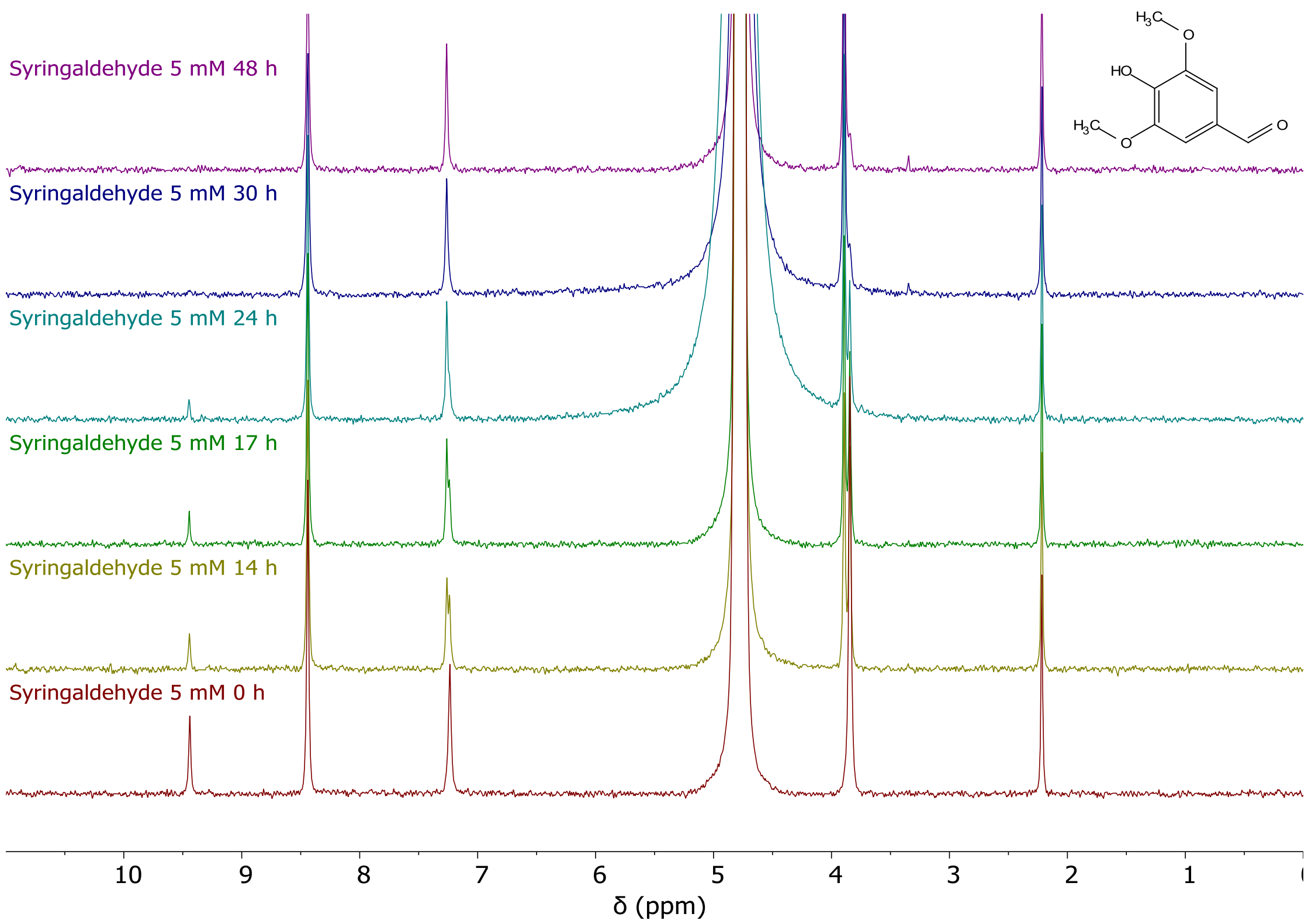

Figure S 10. Stacked ^{1}H NMR spectra for the *E. oleovorans* CECT 5344 biotransformation of syringaldehyde 5 mM at different times.

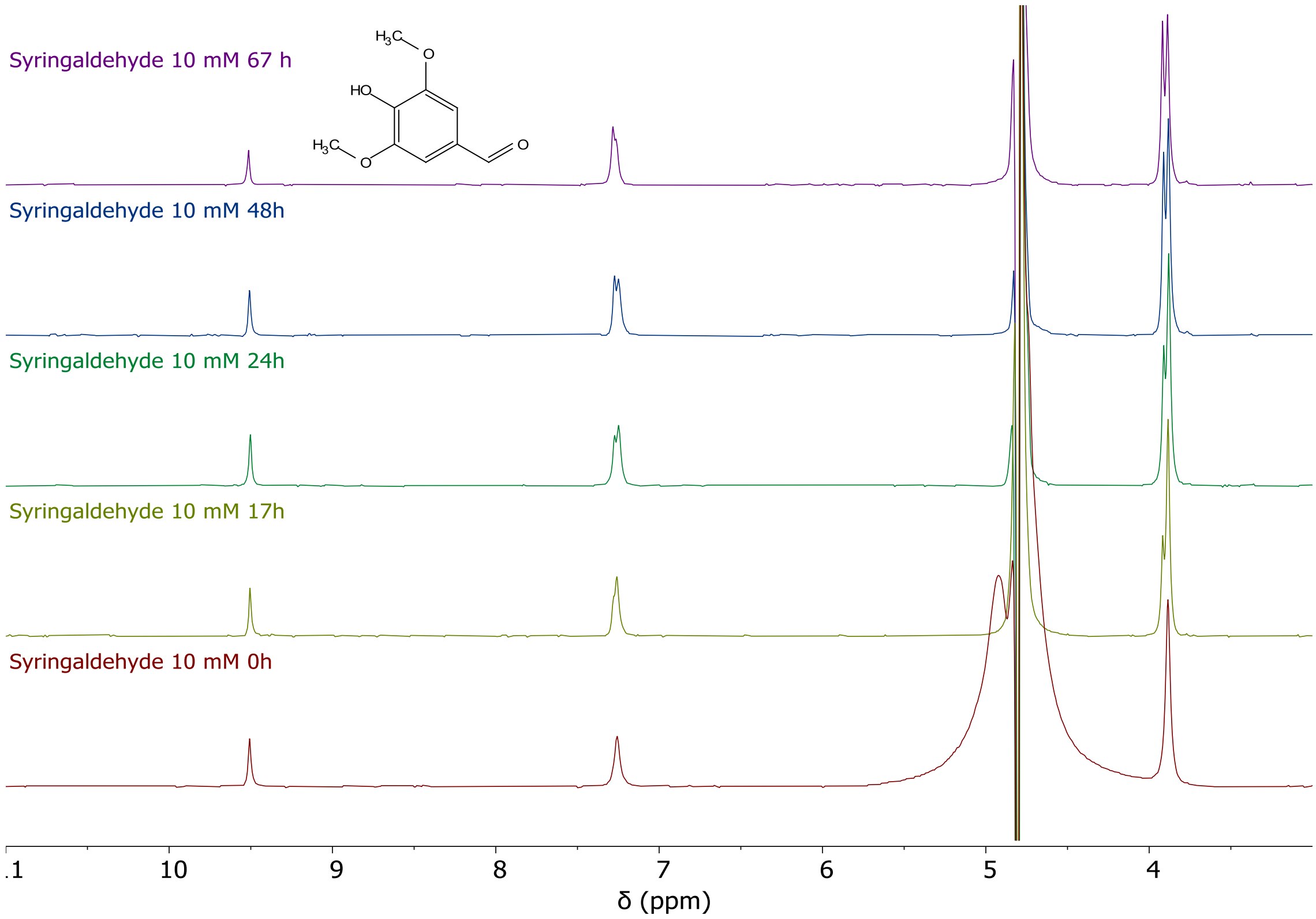

Figure S 11. Stacked ¹H NMR spectra for the *E. oleovorans* CECT 5344 biotransformation of syringaldehyde 10 mM at different times.

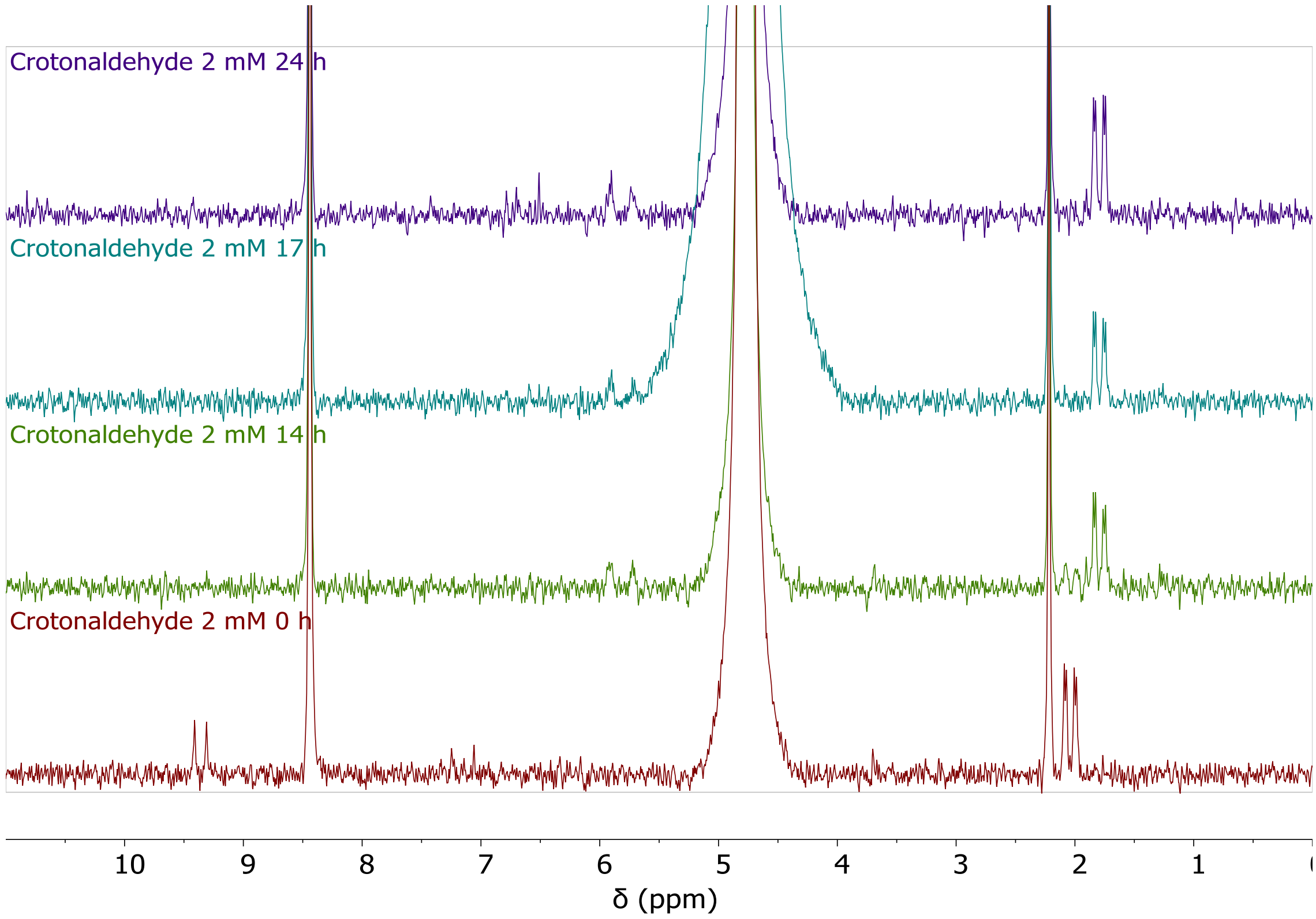

Figure S 12. Stacked ¹H NMR spectra for the *E. oleovorans* CECT 5344 biotransformation of crotonaldehyde 2 mM at different times.

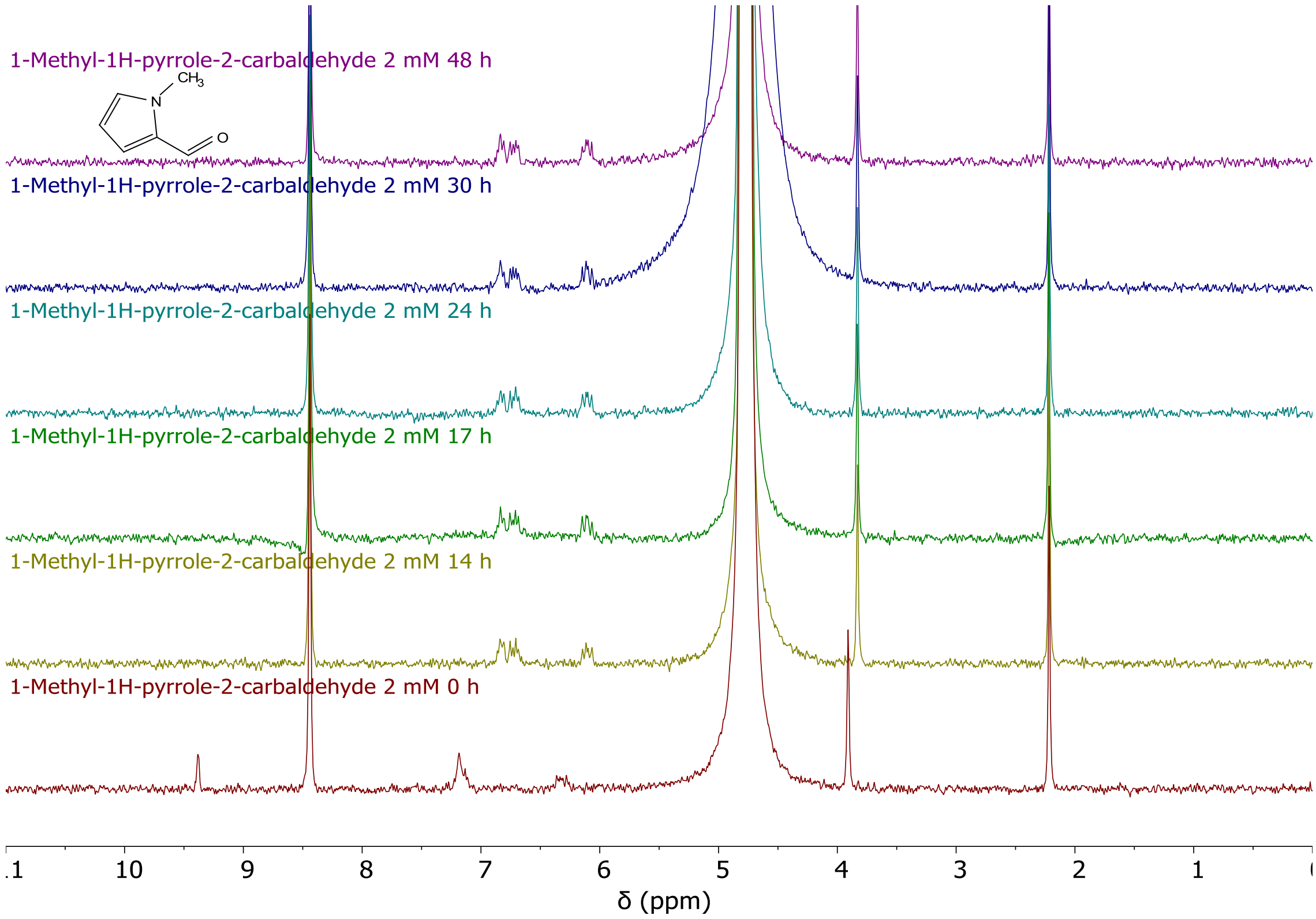

Figure S 13. Stacked ^{1}H NMR spectra for the *E. oleovorans* CECT 5344 biotransformation of 1-methyl-1H-pyrrole-2-carbaldehyde 2 mM at different times.

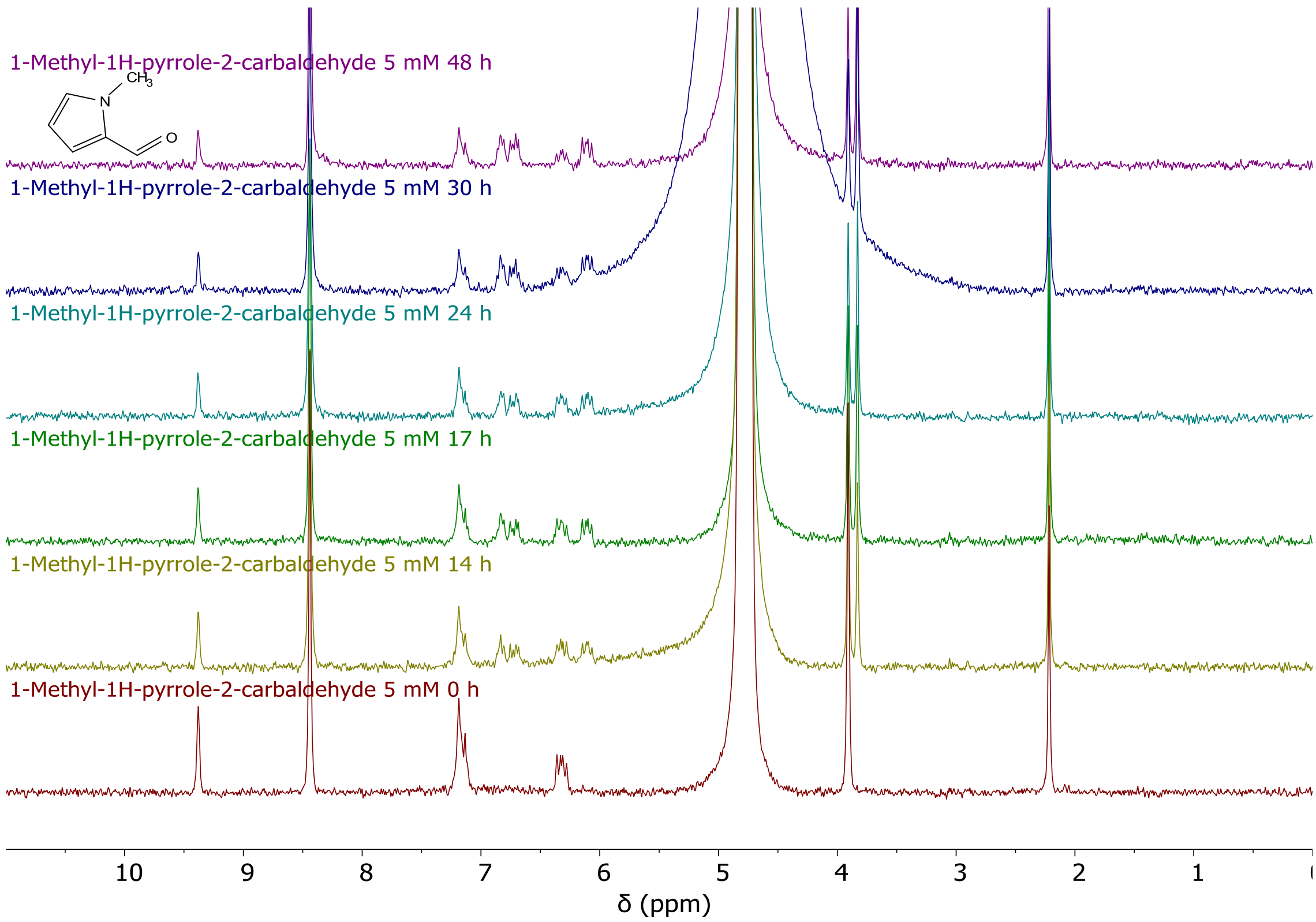

Figure S 14. Stacked ^{1}H NMR spectra for the *E. oleovorans* CECT 5344 biotransformation of 1-methyl-1H-pyrrole-2-carbaldehyde 5 mM at different times.

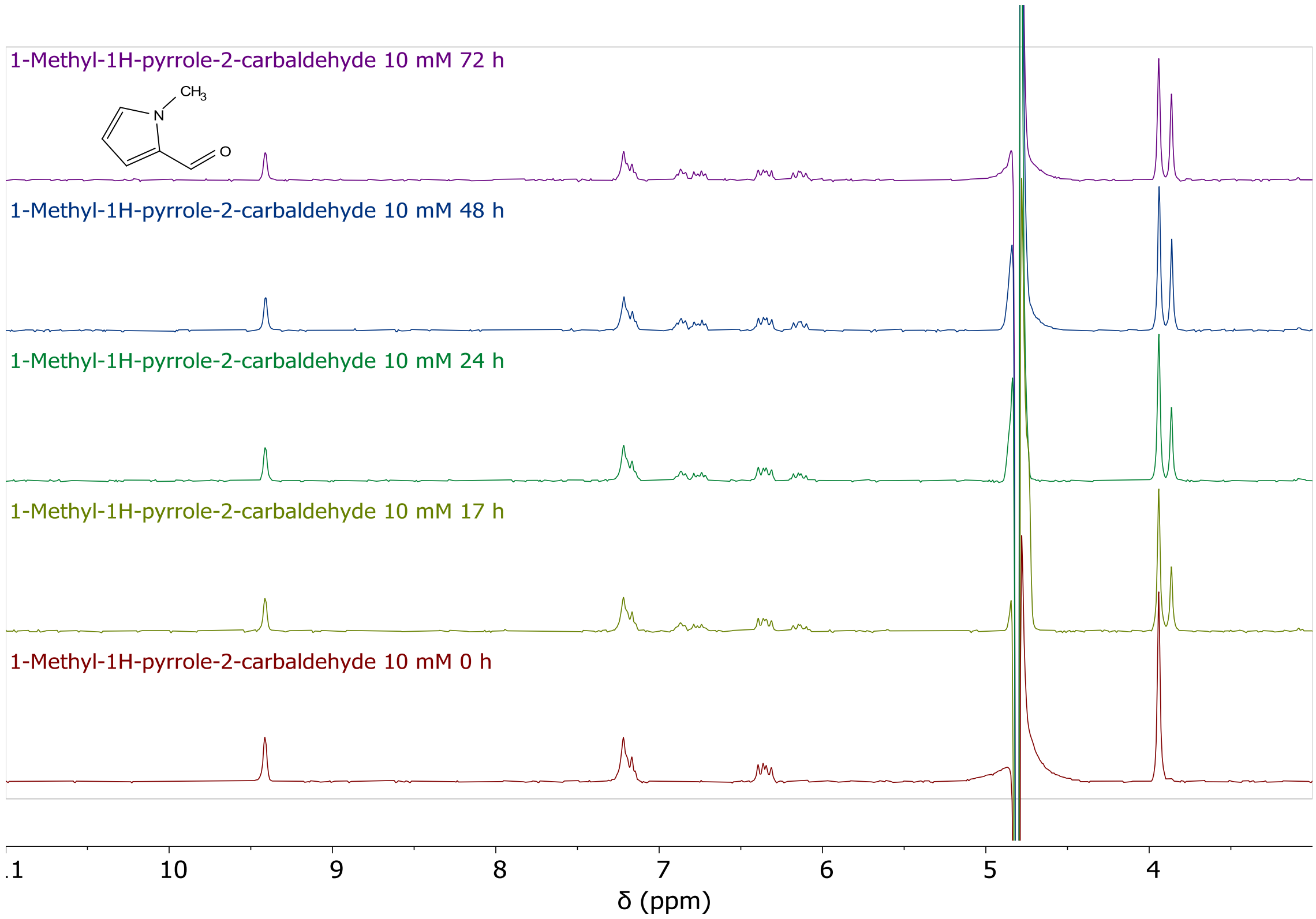

Figure S 15. Stacked ^{1}H NMR spectra for the *E. oleovorans* CECT 5344 biotransformation of 1-methyl-1H-pyrrole-2-carbaldehyde 10 mM at different times.

3. Bacterial viability with and without HMF

Table S 2. Bacterial viability (%) of *E. oleovorans* CECT 5344 in minimal medium in the presence and absence of HMF.[a]

Time (h)	Without HMF x̄ ± SD (CFU/mL)	With HMF x̄ ± SD (CFU/mL)	p-value[b]
0h	100.00 ± 0.00	100.00 ± 0.00	
24h	16.32 ± 21.52	63.26 ± 53.42	0.069
48h	2.94 ± 3.60	25.37 ± 26.56	0.186
72h	6.01 ± 3.78	37.70 ± 38.28	0.321

[a] The viability is expressed as the percentage of viable counts relative to the initial time point and after 24, 48, and 72 hours. Experiments were performed in quadruplicate. The mean (x̄) CFU/mL of the overnight LB cultures was 1.69 × 10^9 CFU/mL. [b] Statistical significance was determined using a two-tailed paired Student's t-test comparing "With HMF" versus "Without HMF" at each time point. Note: While differences did not reach statistical significance ($p > 0.05$), a consistent downward trend was observed in all independent replicates under the 'With HMF' condition, especially at t = 24 h.

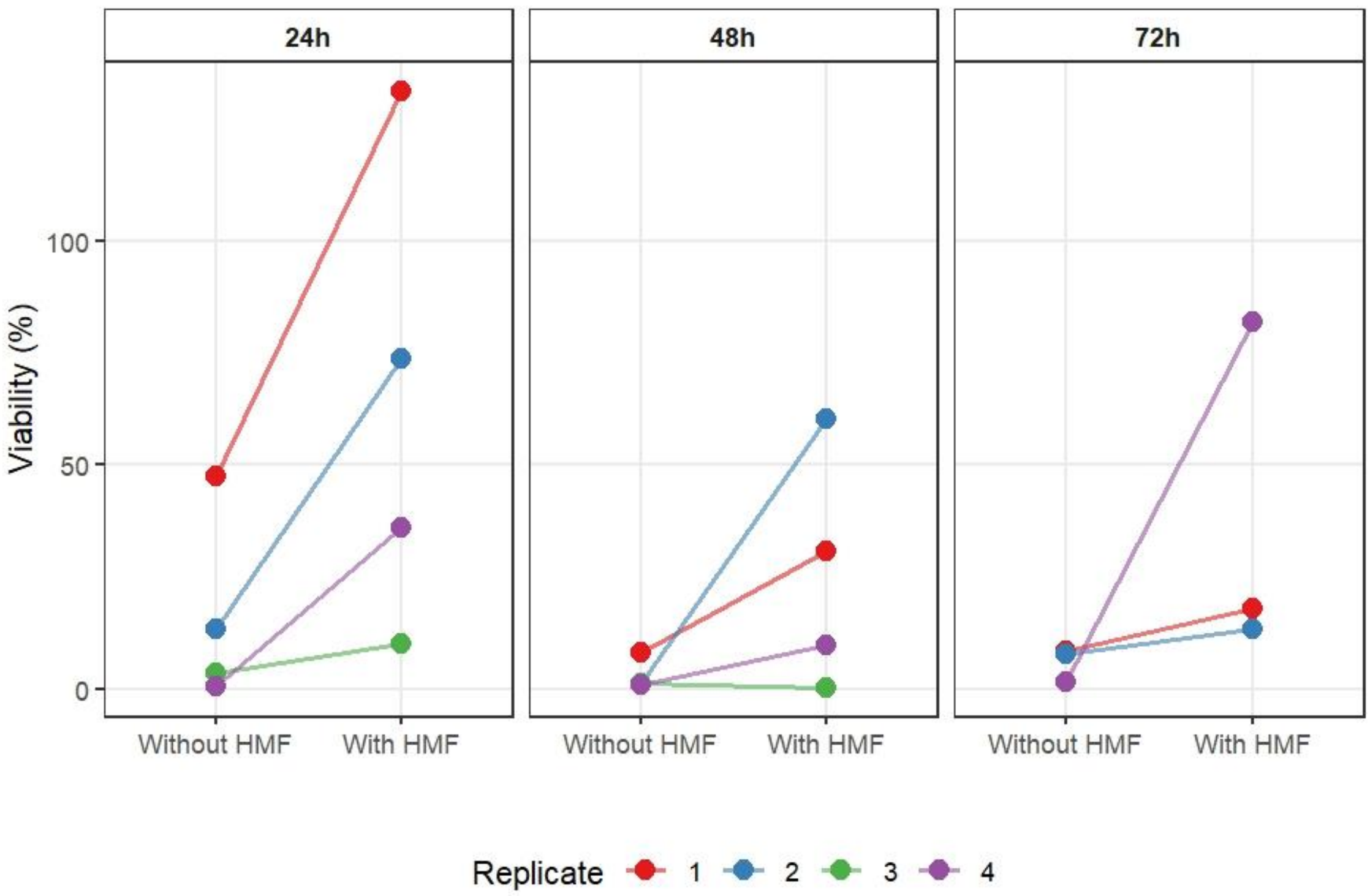

Figure S 16. Bacterial Viability: Individual Experimental Trends. Lines connect samples from the same independent experiment.
